# Carbonylative Synthesis of β‑Trifluoromethylated Heterocyclic Ketones through 1,2-Trifluoromethylation and Minisci Carbonylation of Alkenes

**DOI:** 10.1021/acs.orglett.6c01481

**Published:** 2026-05-12

**Authors:** Ren-Guan Miao, Ru-Han A, Xiao-Feng Wu

**Affiliations:** † Dalian National Laboratory for Clean Energy, 58279 Dalian Institute of Chemical Physics, Chinese Academy of Sciences, 116023 Dalian, Liaoning, China; ‡ Leibniz-Institut für Katalyse e.V., Albert-Einstein-Straße 29a, 18059 Rostock, Germany

## Abstract

The present study details a metal-free catalytic Minisci carbonylation reaction, founded upon a radical polarity reversal strategy. The reaction under scrutiny involves the reaction of an electrophilic trifluoromethyl radical with an electron-rich alkene to generate a nucleophilic alkyl radical, which then undergoes a Minisci carbonylation transformation with various electron-deficient *N*-heteroarenes under a carbon monoxide atmosphere. This method has been demonstrated to be both universal and efficient in the synthesis of trifluoromethyl-substituted *N*-heteroaryl ketones. The experimental results demonstrate that common *N*-heteroaryl substrates, including pyridine, quinoline, and isoquinoline, can all participate in the reaction. In addition, the reaction displays satisfactory compatibility with both terminal and internal alkene substrates.

## Graphical abstract

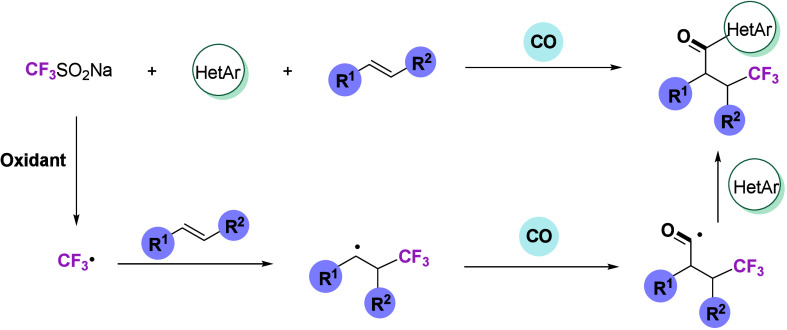

Trifluoromethyl (CF_3_) is one of the most widely used functional groups in medicinal chemistry and agrochemicals. Due to its unique solubility, lipophilicity, metabolic stability, and membrane permeability, it is extensively incorporated into the structural modification of various organic molecules.^1–6^ Among them, β-trifluoromethylated phenylpropionic acid derivatives have been found to act as human peroxisome proliferator-activated receptors.^7^ Consequently, the efficient preparation of β-trifluoromethylcarboxylic acid derivatives has become a research hotspot in the field of organic synthesis. In the context of the rapid advancements witnessed in metal catalysis and photocatalysis technologies, there has been a concomitant development of synthetic methodologies. Researchers have successfully prepared β-trifluoromethyl carboxylic acid derivatives through asymmetric hydrogenation reactions of α- or β-trifluoromethylacrylic acid, with the catalysis provided by rhodium.^8^ The synthesis of β-trifluoromethyl alkyl aryl ketones has been achieved through the use of a synergistic catalytic system comprising a nitrogen heterocyclic carbene and photoredox and Langlos’ reagent as a trifluoromethyl donor.^9^ The efficient preparation of β-trifluoromethyl carboxylic acid derivatives has also been achieved through transition metal catalytic carbonylation and other strategies, using Togni II as a trifluoromethyl source.^10^ Furthermore, a new method for preparing β-trifluoromethyl thioesters under visible light induction has been developed, using Langlos’ reagent as a trifluoromethyl source (Scheme 1A).^11^

**Scheme 1:**
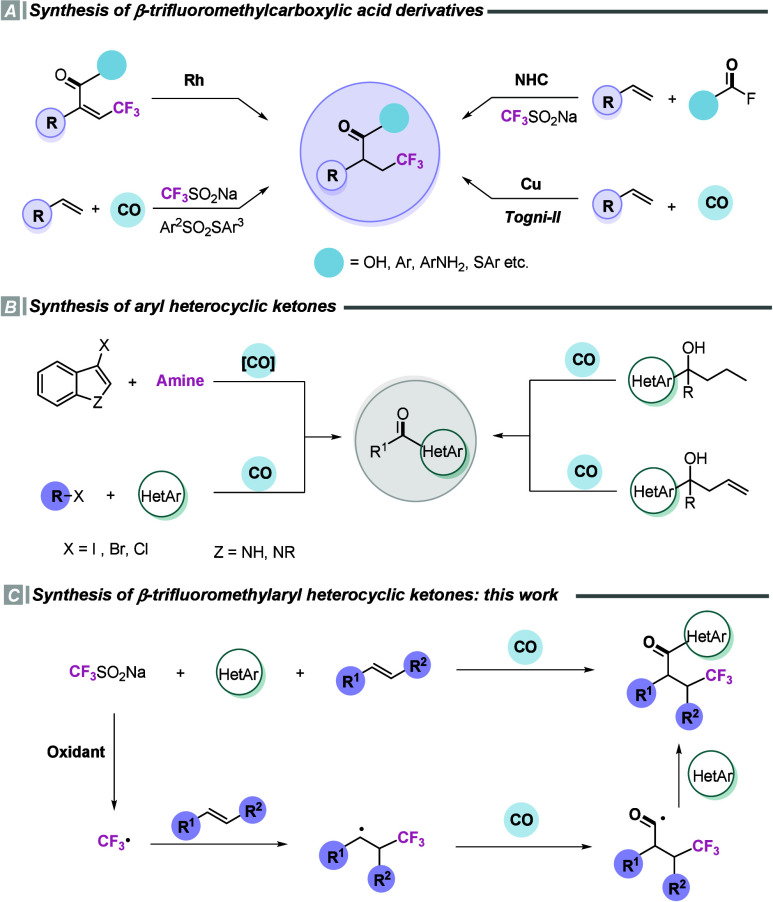
Studies on Minisci Carbonylation of Alkenes

Nitrogen heterocycles are prevalent structural units in natural products and the core framework for synthesizing bioactive molecules. It is notable that a significant proportion of commercially available specialty small molecule drugs contain this structure, and they are also widely used as ligands, organic catalysts, and synthetic directing groups. The development of synthetic strategies for their derivatives has historically been a major focus of research in the field of organic synthesis.^12–15^ The carbonyl group is a pivotal functional group in natural products, pharmaceuticals, functional materials, and fine chemicals. A significant proportion of the world’s top 100 best-selling small molecule drugs have been found to contain carbonyl fragments, with the proportion ranging from 80% to 90%.^16^ The efficient and precise construction of this functional group is one of the core issues in modern synthetic chemistry and drug development. Carbonylation reactions utilizing CO as the carbonyl source have emerged as the prevailing strategy for synthesizing a range of carbonyl-containing compounds. This approach is notable for its high atom economy, straightforward process, and broad substrate applicability. Furthermore, they represent a pivotal conduit for the high-value transformation of C1 resources. Consequently, the synthesis of aryl heterocyclic ketones holds considerable research and application value. In recent years, researchers have developed a variety of green synthetic methods for producing heterocyclic ketones, including palladium-catalyzed carbonylation of heterocycles via C–H functionalization reported in 2015, palladium-catalyzed amino carbonylation of halogenated heteroarenes reported in 2017, and visible-light-induced heterocyclic migration carbonylation reaction reported in 2024–2025 (Scheme 1B).^17–22^ However, the carbonylative synthesis of β-trifluoromethyl aryl heterocyclic ketones still faces many challenges.

Minisci reactions represent a promising synthetic strategy, capable of introducing a wide range of C­(sp^3^) alkyl motifs into N-heterocyclic skeletons, with high efficiency in the construction of C­(sp^3^)–C­(sp^3^) and C­(sp^3^)–C­(sp^2^) covalent bonds.^23,24^ Nevertheless, the control of the reaction efficiency and regioselectivity in the introduction of trifluoromethyl groups into electron-deficient N-heteroaromatics via Minisci reactions remains challenging.^25^ In consideration of the particular nucleophilicity requirements of the carbon-centered radical in the coupling process with electron-deficient heteroaromatics, it is hypothesized that Minisci carbonylation can be achieved through a cascade strategy involving radical polarity reversal. This strategy has been demonstrated to induce the addition reaction of electrophilic trifluoromethyl radicals to alkenes with high chemoselectivity and regioselectivity, further generating acyl radicals under a carbon monoxide atmosphere. These nucleophilic radicals have been shown to selectively couple with electrophilic heteroaromatics, ultimately yielding trifluoromethyl-substituted N-heteroaromatic ketone derivatives. The present study proposes the use of the cost-effective Langlois reagent (CF_3_SO_2_Na) as a trifluoromethyl radical source to generate trifluoromethyl radicals through oxidation.^26,27^ Following the addition of radicals to unactivated alkenes by the radicals, acyl radicals are generated in a carbon monoxide atmosphere. These then couple with electrophilic heteroaromatics to achieve a metal-free Minisci carbonylation reaction (Scheme 1C).

In accordance with the aforementioned reaction design, the utilization of hex-1-ene (**1a**), CF_3_SO_2_Na (**2a**), and 4-methylquinoline (**3a**) as template substrates enabled the employment of *tert*-butyl peroxyacetate (TBPA) as the oxidant. The reaction was conducted at ambient temperature under a 40 bar CO atmosphere, with DMSO serving as the solvent. This initial procedure resulted in the attainment of the target product, in 35% yield (Table 1, entry 1). Subsequently, oxidant screening was conducted, and the results showed that di-*tert*-butyl peroxide (DTBP) failed to generate the target product, whereas *tert*-butyl hydrogen peroxide (TBHP) had a 30% yield of a product. Consequently, *tert*-butyl peroxyacetate (TBPA) was identified as the optimal oxidant (Table 1, entries 2–3). Following the optimization of the reaction substrate ratio, a product yield of 45% was achieved (Table 1, entry 4). However, in the solvent screening experiment, a significant decrease in yield was observed after changing the solvent, and the target compound was not detected when THF was used as the solvent (Table 1, entries 5–7). Adjustments to the oxidant dosage were made, and it was established that a target product yield of 50% was achieved with 3.5 equiv of TBPA (Table 1, entry 8). Subsequent optimization of the trifluoroacetic acid (TFA) dosage, with six equivalents of TFA, resulted in a 60% increase in product yield (Table 1, entry 9). A decrease in the CO pressure to 30 bar was observed to result in a consequent yield decrease to 52% (Table 1, entry 10). However, the removal of the oxidant did not result in the detection of target product formation within the system (Table 1, entry 11). In summary, following the screening of a series of reaction conditions, the optimal reaction conditions were determined to be as follows: **1a** (0.3 mmol), **2a** (0.2 mmol), and **3a** (0.1 mmol) as substrates, TBPA (3.5 equiv) as oxidant, TFA (6 equiv) as additive, DMSO (1 mL) and H_2_O (0.5 mL) as mixed solvents, and reaction at 40 bar CO atmosphere and room temperature. Under such conditions, the separation yield of the target product can reach 60%. During the optimization process, noncarbonylation was the main side reaction, which is reasonable for the low obtained yield.

**Table 1:**
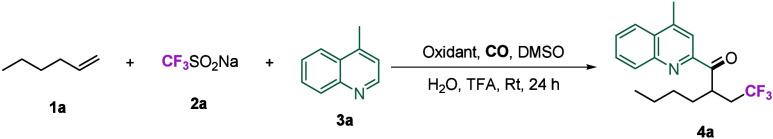
Optimization of the Reaction Conditions^a^

Entry	Oxidant	Solv.	Yield **4a** (%)
1	TBPA	DMSO	35
2	DTBP	DMSO	N.D.
3	TBHP	DMSO	30
4^b^	TBPA	DMSO	45
5^b^	TBPA	DMF	17
6^b^	TBPA	MeCN	13
7^b^	TBPA	THF	N.D.
8^b^ ^,^ ^c^	TBPA	DMSO	50
9^b^ ^,^ ^c^ ^,^ ^d^	TBPA	DMSO	60
10^b^ ^,^ ^c^ ^,^ ^d^ ^,^ ^e^	TBPA	DMSO	52
11^b^ ^,^ ^c^ ^,^ ^d^ ^,^ ^e^ ^,^ ^f^	TBPA	DMSO	N.D.

a Reaction conditions: **1a** (0.2 mmol), **2a** (0.3 mmol), **3a** (0.1 mmol), TBPA (3 equiv), TFA (2 equiv), DMSO (1 mL), H_2_O (0.5 mL), CO (40 bar), rt, 24 h. Isolated yields

b **1a** (0.3 mmol), **2a** (0.2 mmol), and **3a** (0.1 mmol).

c TBPA (3.5 equiv).

d TFA (6 equiv).

e CO (30 bar).

f Without TBPA. TBPA = *tert*-butyl peroxyacetate.

Following the optimization of the reaction conditions, the universality of the reaction for heterocyclic substrates was investigated further, the results of which are summarized in Scheme 2. Under the aforementioned optimal conditions, a variety of substituted heterocyclic substrates have been shown to participate in the reaction in a smooth and efficient manner, thereby yielding the desired products. In the initial phase of the experiment, electron-donating-substituted quinolines were selected as substrates. These included tetramethylquinoline and tetramethoxyquinoline, both of which yielded the target products in satisfactory yields (**4a** and **4b**). Subsequently, an investigation was conducted into electron-withdrawing-substituted quinolines. Irrespective of whether the halogenation was monohalogenated or polyhalogenated, the reaction proceeded in a satisfactory manner, and the products were obtained in satisfactory yields (**4c**–**4f**). In the case of the substrate being aldehyde (**4g**), cyano (**4h**), or ester (**4i**) substituted quinolines, the yield of the target product was moderate. The decline in yield is hypothesized to be attributable to the presence of strong electron-withdrawing groups, which significantly reduce the electron density of the quinoline ring, thereby impacting reactivity. Subsequently, quinoline substrates with varying substituents at the 2-position (methyl and phenyl) were also examined, resulting in the successful synthesis of the target products in moderate to good yields (**4j**–**4k**). Subsequent to the alteration of the substrate to 6-chloroisoquinoline, the reaction continued, yielding the target product (**4l**) in a moderate yield. Furthermore, we attempted to utilize pyridines with electron-donating (*tert*-butyl) and electron-withdrawing (cyano) groups at the 4-position as substrates. This approach resulted in the synthesis of the desired target products in moderate yields, suggesting that pyridine substrates are also compatible with this reaction system.

**Scheme 2:**
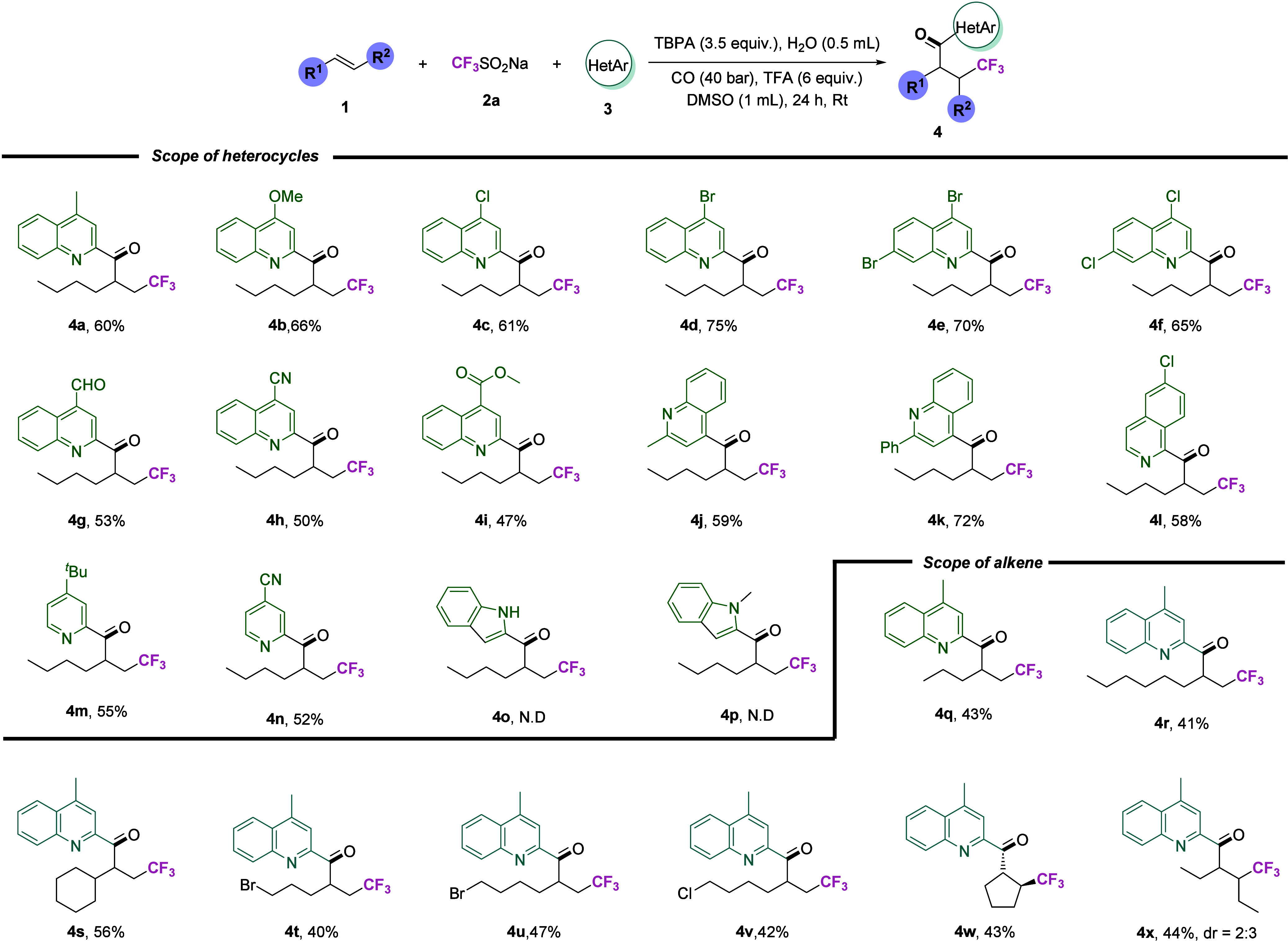
Substate Scope of Heterocycles and Unactivated Alkenes^a^

a Reaction conditions: **1a** (0.3 mmol), **2a** (0.2 mmol), **3a** (0.1 mmol), TBPA (3.5 equiv), TFA (6 equiv), DMSO (1 mL), H_2_O (0.5 mL), CO (40 bar), rt, 24 h. Isolated yields.

However, no target product was detected (**4o**–**4p**) when indoles were utilized as substrates, which might be due to the relatively increased difficulty on the rearomatization of the intermediate under our conditions. Acridine was tested as well but led to no detectable target product. Additionally, both 2- and 4-acylated products were detected when 4-unsubstituted quinolines were applied. In addition, the universality of this reaction with alkene substrates was investigated. First, we tested alkenes with different alkyl chain lengths, selecting pent-1-ene, oct-1-ene, and vinylcyclohexane as substrates, all of which yielded the target products in moderate yields (**4q**–**4s**). Subsequently, we attempted various halogen-substituted alkenes, and the reactions proceeded smoothly, also yielding products in moderate yields (**4t**–**4v**). We further tested cyclic alkene and obtained the target product in a 43% yield (**4w**); the test of internal alkene also had a 44% yield, indicating that the reaction system has good compatibility with internal alkenes.

In order to further investigate the reaction mechanism, a series of control experiments were designed and conducted. The results of these experiments are shown in Scheme 3. Initially, we incorporated TEMPO under standard reaction conditions to ascertain whether the reaction proceeded via a radical mechanism. No target product was detected, and high-resolution mass spectrometry (HRMS) did not identify any radical-capturing intermediates, suggesting that these intermediates may have a poor stability. Subsequently, we replaced TEMPO with BHT under standard conditions, detecting only a small amount of the target product, and HRMS again failed to detect any related radical capturing intermediates. However, when 1,1-diphenylethene (DPE) was added, the CF_3_ radical could be captured and detected. Control experiments conducted indicate that the reaction most likely proceeds via a radical mechanism. Furthermore, an increase in the substrate amount to 1 mmol was made, which ultimately resulted in a 45% yield of the target product.

**Scheme 3:**
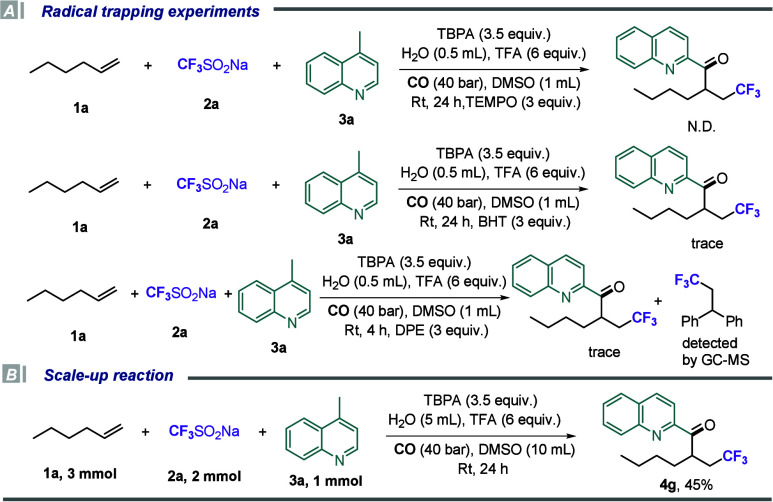
Control Experiments and Scale-Up Reaction

Drawing upon the findings of our experiments, we propose a possible reaction mechanism (Scheme 4). First, CF_3_SO_2_Na reacts with an oxidant to generate a trifluoromethanesulfonyl radical **B**. Subsequently, the trifluoromethanesulfonyl radical eliminates sulfur dioxide, thereby yielding trifluoromethyl radical **C**. Subsequently, the trifluoromethyl radical selectively combines with an alkene to generate nucleophilic alkyl radical **D**. In the presence of carbon monoxide, **D** undergoes carbonylation to yield intermediate **E**, which immediately reacts with an electron-deficient *N*-heteroaromatic hydrocarbon and, through oxidation, finally yields the target product **H**.

**Scheme 4:**
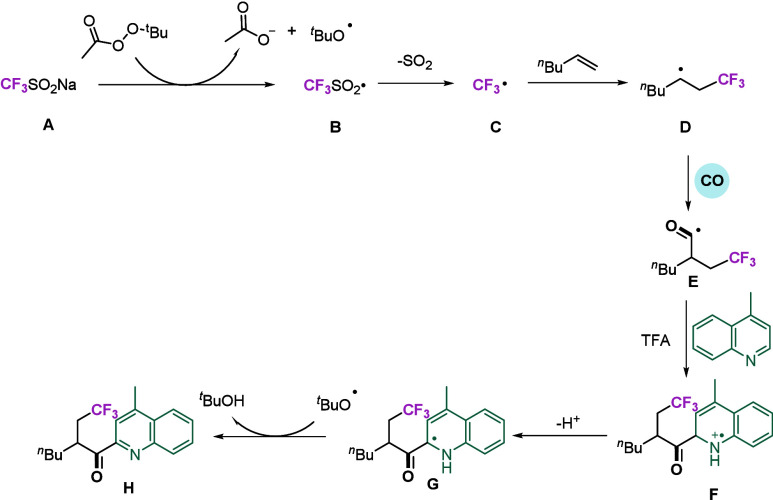
Proposed Mechanism

In summary, we report here a Minisci carbonylation reaction mediated by a trifluoromethyl radical and based on a radical polarity reversal strategy. This reaction does not necessitate the use of a transition-metal catalyst. The electrophilic trifluoromethyl radical subsequently adds to an electron-rich alkene, resulting in the generation of a nucleophilic alkyl radical. This radical then undergoes a Minisci carbonylation reaction with various electron-deficient *N*-heteroaromatics under a carbon monoxide atmosphere. This provides a general and efficient new route for the synthesis of trifluoromethyl-substituted *N*-heteroaromatic ketones. As demonstrated, *N*-heteroaromatic substrates, including pyridine, quinoline, and isoquinoline, have been shown to participate reliably in the reaction within this system. Furthermore, the reaction was observed to exhibit excellent compatibility with both terminal and internal alkene substrates.

## Supplementary Material



## Data Availability

The data underlying this study are available in the published article and its Supporting Information.

## Notes

Experimental details, characterization data, and NMR spectra (PDF)
