# Supplementary material for: Carbonylative Synthesis of β‑Trifluoromethylated Heterocyclic Ketones through 1,2-Trifluoromethylation and Minisci Carbonylation of Alkenes
Source: Org Lett. 2026 May 12;28(20):6371–5. doi: 10.1021/acs.orglett.6c01481 (PMC13200246; doi:10.1021/acs.orglett.6c01481)

Supporting Information

Carbonylative Synthesis of *β*-Trifluoromethylated Heterocyclic Ketones through 1,2-Trifluoromethylation and Minisci Carbonylation of Alkenes

Ren-Guan Miao,[a,b] Ru-Han A,[a,b] and Xiao-Feng Wu[a,b]*

[a] Dalian National Laboratory for Clean Energy, Dalian Institute of Chemical Physics, Chinese Academy of Sciences, 116023 Dalian, Liaoning, China, E-mail: xwu2020@dicp.ac.cn

[b] Leibniz-Institut für Katalyse e.V., Albert-Einstein-Straße 29a, 18059 Rostock, Germany

Contents

1. General information 1
2. Optimization of the reaction conditions 2
3. General procedure for the synthesis of β-trifluoromethyl heterocyclic ketones.....6
4. Control experiments 7
5. A scale-up reaction 7
6. Characterization data of the corresponding product 8
7. The NMR spectrum 20

1. General information

Unless otherwise noted, all reactions were carried out under N_2. All reagents were from commercial sources (Energy Chemical; Redia; Laajoo) and used as received without further purification. All solvents were dried by standard techniques and distilled prior to use. Column chromatography was performed on silica gel (200-300 meshes) using petroleum ether (bp. 60~90 °C) as eluent. ^{1}H and ^{13}C NMR spectra were taken on 400 MHz or 700 MHz instruments and spectral data were reported in ppm relative to tetramethylsilane (TMS) as the internal standard and $CDCl_3$ (^{1}H NMR δ 7.26, ^{13}C NMR δ 77.16) as solvent. All coupling constants (*J*) are reported in Hz with the following abbreviations: s = singlet, d = doublet, dd = double doublet, ddd = double doublet of doublets, t = triplet, dt = double triplet, q = quartet, m = multiplet, br = broad. All reactions were monitored by GC-FID or NMR analysis. HRMS data was obtained with Micromass HPLC-Q-TOF mass spectrometer (ESI-TOF) or Agilent 6540 Accurate-MS spectrometer (Q-TOF). Because of the high toxicity of carbon monoxide, all the reactions should be performed in an autoclave. The laboratory should be well-equipped with a CO detector and alarm system.

2. Optimization of the reaction conditions

Table S1. Optimization of Oxidant

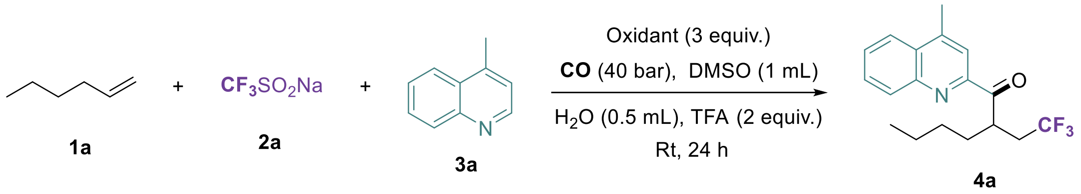

Entry	**Oxidant**	Yield (%)
1	TBPA	35
2	LPO	N.D.
3	DTBP	N.D.
4	TBPB	N.D.
5	CHP	17
6	BPO	N.D.
7	TBHP	30
8	$K_2S_2O_8$	trace
9	$Na_2S_2O_8$	trace

Reaction conditions: **1a** (0.2 mmol), **2a** (0.3 mmol), **3a** (0.1 mmol), Oxidant (3 equiv.), TFA (2 equiv.), DMSO (1 mL), H_2O (0.5 mL), CO (40 bar), Rt, 24 h. Isolated yields.

Table S2. Optimization of substrate ratio

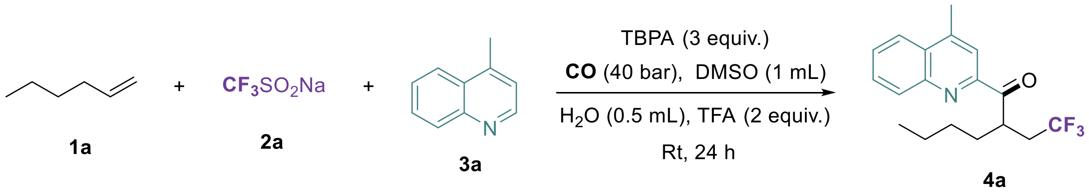

Entry	**1a : 2a : 3a (mmol)**	Yield (%)
1	0.1 : 0.3 : 0.1	23
2	0.1 : 0.3 : 0.15	25
3	0.1 : 0.3 : 0.2	18
4	0.1 : 0.3 : 0.3	trace
5	0.15 : 0.3 : 0.1	30

6	0.25 : 0.3 : 0.1	39
7	0.3 : 0.3 : 0.1	41
8	0.35 : 0.3 : 0.1	39
9	0.3 : 0.15 : 0.1	44
10	0.3 : 0.2 : 0.1	45
11	0.3 : 0.25 : 0.1	43
12	0.3 : 0.35 : 0.1	40

Reaction conditions: **1a** (x mmol), **2a** (y mmol), **3a** (z mmol), TBPA (3 equiv.), TFA (2 equiv.), DMSO (1 mL), H_2O (0.5 mL), CO (40 bar), Rt, 24 h. Isolated yields.

Table S3. Optimization of H_2O dosage

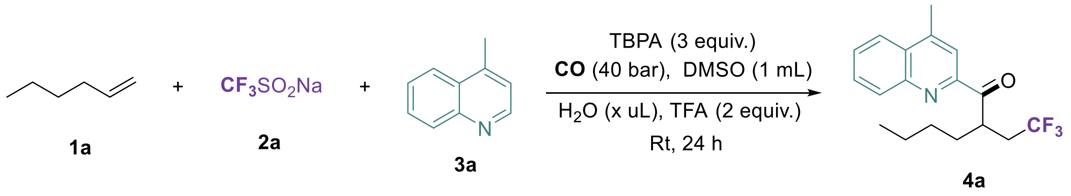

Entry	H_2O (uL)	Yield (%)
1	0	N.D.
2	10	trace
3	50	trace
4	100	trace
5	200	trace
6	300	trace
7	700	36
8	1000	13

Reaction conditions: **1a** (0.3 mmol), **2a** (0.2 mmol), **3a** (0.1 mmol), TBPA (3 equiv.), TFA (2 equiv.), DMSO (1 mL), H_2O, CO (40 bar), Rt, 24 h. Isolated yields.

Table S4. Optimization of solvent

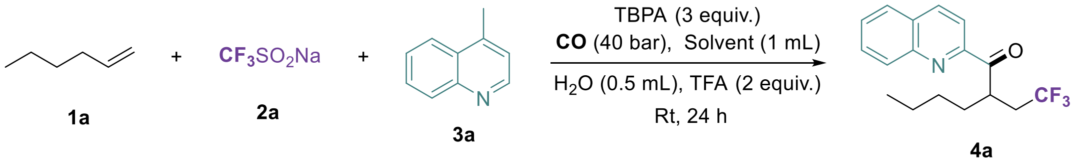

Entry	Solvent (x mL)	Yield (%)
1	DMF	17
2	DMAc	11
3	NMP	N.D.
4	$PhCF_3$	N.D.
5	MeCN	13
6	THF	N.D.
7	EA	trace
8	HFIP	trace

Reaction conditions: **1a** (0.3 mmol), **2a** (0.2 mmol), **3a** (0.1 mmol), TBPA (3 equiv.), TFA (2 equiv.), Solvent (1 mL), H_2O (0.5 mL), CO (40 bar), Rt, 24 h. Isolated yields.

Table S5. Optimization of TBPA dosage

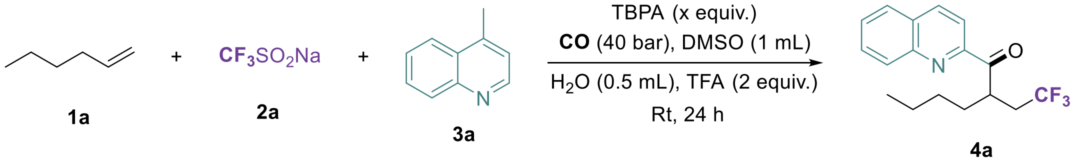

Entry	TBPA (x equiv.)	Yield (%)
1	1	31
2	1.5	39
3	2	43
4	2.5	45
5	3.5	50
6	4	48

Reaction conditions: **1a** (0.3 mmol), **2a** (0.2 mmol), **3a** (0.1 mmol), TBPA (x equiv.), TFA (2 equiv.), DMSO (1 mL), H_2O (0.5 mL), CO (40 bar), Rt, 24 h. Isolated yields.

Table S6. Optimization of TFA dosage

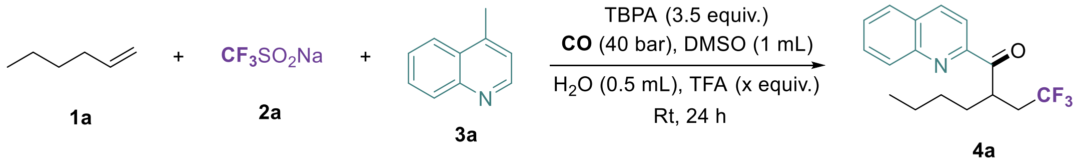

Entry	TFA (x equiv.)	Yield (%)
1	0	N.D.
2	0.5	42
3	1	25
4	3	50
5	4	53
6	5	49
7	6	60

Reaction conditions: **1a** (0.3 mmol), **2a** (0.2 mmol), **3a** (0.1 mmol), TBPA (3.5 equiv.), TFA (x equiv.), DMSO (1 mL), H_2O (0.5 mL), CO (40 bar), Rt, 24 h. Isolated yields.

Table S7. Optimization of CO pressure

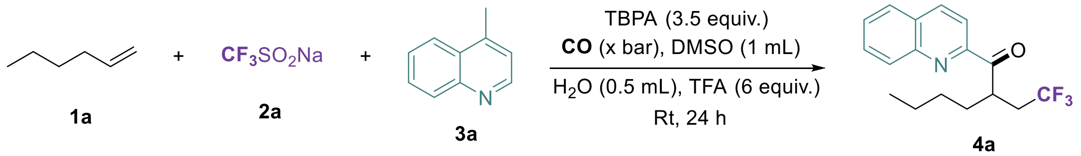

Entry	**CO** (bar)	Yield (%)
1	20	41
2	30	52
3	50	48

Reaction conditions: **1a** (0.3 mmol), **2a** (0.2 mmol), **3a** (0.1 mmol), TBPA (3.5 equiv.), TFA (6 equiv.), DMSO (1 mL), H_2O (0.5 mL), CO (x bar), Rt, 24 h. Isolated yields.

3. General procedure for the synthesis of β-trifluoromethyl heterocyclic ketones

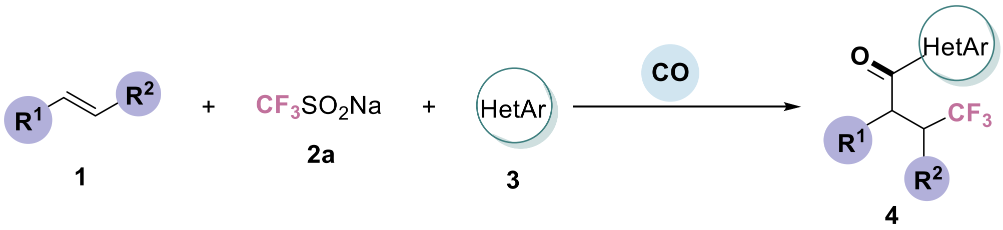

A 4 mL screw-cap vial was charged with **1** (0.3 mmol; 3 equiv.), **2a** (0.2 mmol; 2 equiv.), **3** (0.1 mmol; 1 equiv.), TBPA (0.35 mmol; 3.5 equiv.) (tert-butyl peroxyacetate, CAS: 107-71-1), TFA (0.6 mmol; 6 equiv.), H_2O (0.5 mL) and an oven-dried stirring bar. The vial was closed with a Teflon septum and cap and connected to the atmosphere via a needle. Then DMSO (1 mL) was added with a syringe under N_2 atmosphere. The closed autoclave was flushed two times with nitrogen (~ 10 bar), and a pressure of 40 bar CO were charged. The reaction mixture was stirred at room temperature for 24 hours. After the reaction, the pressure was released carefully. The mixture was concentrated under vacuum. The crude product was purified by column chromatography (PE/EA = 50/1) on silica gel to afford the corresponding products.

4. Control experiments

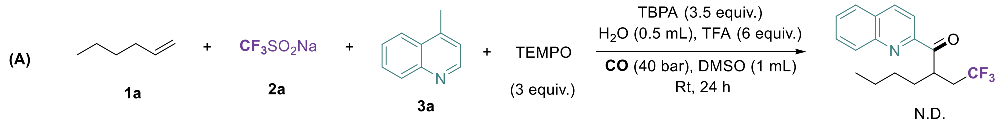

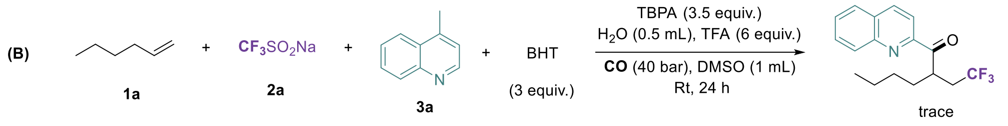

A 4 mL screw-cap vial was charged with **1a** (0.3 mmol), **2a** (0.2 mmol), **3a** (0.1 mmol), TBPA (3.5 equiv.), TFA (6 equiv.), H_2O (0.5 mL), radical scavenger (3 equiv.) and an oven-dried stirring bar. The vial was closed with a Teflon septum and cap and connected to the atmosphere via a needle. Then DMSO (1 mL) was added with a syringe under N_2 atmosphere. The closed autoclave was flushed two times with nitrogen (~ 10 bar), and a pressure of 40 bar CO were charged. The reaction mixture was stirred at room temperature for 24 hours.

5. A scale-up reaction

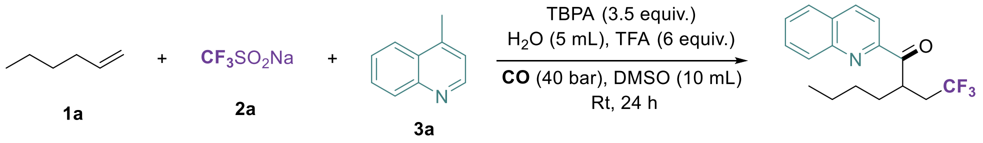

A 50 mL round bottom flask was charged with **1a** (3 mmol), **2a** (2 mmol), **3a** (1 mmol), TBPA (3.5 mmol; 3.5 equiv.), TFA (6 mmol; 6 equiv.), H_2O (5 mL) and an oven-dried stirring bar. The vial was closed with a rubber stopper and connected to the atmosphere via a needle. Then DMSO (10 mL) was added with a syringe under N_2 atmosphere. The closed autoclave was flushed two times with nitrogen (~ 10 bar), and a pressure of 40 bar CO were charged. The reaction mixture was stirred at room temperature for 24 hours. After the reaction, the pressure was released carefully. The mixture was concentrated under vacuum. The crude product was purified by column chromatography (PE/EA = 50/1) on silica gel to afford the corresponding products **4a** as a yellow liquid (145.4 mg, 45%).

6. Characterization data of the corresponding product

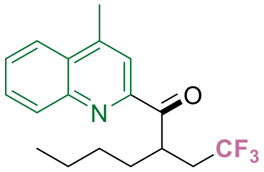

1-(4-methylquinolin-2-yl)-2-(2,2,2-trifluoroethyl)hexan-1-one

The reaction solution was processed as general experimental procedure to afford the corresponding product **4a** as a yellow liquid (19.4 mg, 60%; PE/EA = 50/1).

^{1}H NMR (400 MHz, $CDCl_3$) δ 8.26 – 8.14 (m, 1H), 8.04 (d, J = 8.3 Hz, 1H), 7.98 (s, 1H), 7.78 (t, J = 7.6 Hz, 1H), 7.68 (t, J = 7.6 Hz, 1H), 4.83 – 4.67 (m, 1H), 3.06 – 2.86 (m, 1H), 2.77 (s, 3H), 2.47 – 2.18 (m, 1H), 1.93 – 1.75 (m, 1H), 1.64 (dd, J = 14.1, 7.1 Hz, 1H), 1.38 – 1.27 (m, 4H), 0.85 (t, J = 6.7 Hz, 3H).

^{13}C NMR (101 MHz, $CDCl_3$) δ 203.1, 151.5, 147.1, 145.5, 131.5, 129.7, 129.6, 128.5, 126.9 (q, $J_{C\text{-}F}$ = 277.1 Hz), 123.8, 119.1, 37.9 (q, $J_{C\text{-}F}$ = 2.2 Hz), 34.6 (q, $J_{C\text{-}F}$ = 28.4 Hz), 32.3, 28.8, 22.4, 18.9, 13.8.

^{19}F NMR (376 MHz, $CDCl_3$) δ -64.7.

HRMS (ESI-TOF): calcd for $[M+H]^+$ $C_{18}H_{21}F_3NO^+$ 324.1570, found: 324.1559.

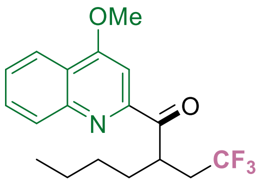

1-(4-methoxyquinolin-2-yl)-2-(2,2,2-trifluoroethyl)hexan-1-one

The reaction solution was processed as general experimental procedure to afford the corresponding product **4b** as a yellow liquid (22.4 mg, 66%; PE/EA = 20/1).

^{1}H NMR (400 MHz, $CDCl_3$) δ 8.23 (d, J = 8.3 Hz, 1H), 8.14 (d, J = 8.5 Hz, 1H), 7.76 (t, J = 7.6 Hz, 1H), 7.61 (t, J = 7.6 Hz, 1H), 7.52 (s, 1H), 4.80 – 4.72 (m, 1H), 4.12 (s, 3H), 2.99 – 2.84 (m, 1H), 2.39 – 2.26 (m, 1H), 1.90 – 1.80 (m, 1H), 1.67 – 1.57 (m, 1H), 1.37 – 1.29 (m, 4H), 0.86 (t, J = 6.7 Hz, 3H).

^{13}C NMR (101 MHz, CDCl$_3$) δ 203.0, 163.2, 153.2, 148.2, 130.4, 130.2, 127.8, 126.9 (q, J_{C-F} = 276.8 Hz), 122.7, 121.9, 97.2, 56.0, 37.9 (q, J_{C-F} = 1.8 Hz), 34.7 (q, J_{C-F} = 28.4 Hz), 32.4, 28.8, 22.4, 13.8.

^{19}F NMR (376 MHz, CDCl$_3$) δ -64.7.

HRMS (ESI-TOF): calcd for $[M+H]^+$ $C_{18}H_{21}F_3NO_2^+$ 340.1519; Found: 340.1518.

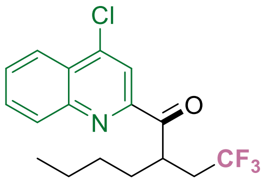

1-(4-chloroquinolin-2-yl)-2-(2,2,2-trifluoroethyl)hexan-1-one

The reaction solution was processed as general experimental procedure to afford the corresponding product **4c** as a yellow liquid (20.9 mg, 61%; PE/EA = 50/1).

^{1}H NMR (400 MHz, CDCl$_3$) δ 8.25 – 8.16 (m, 2H), 8.15 (s, 1H), 7.78 (t, J = 7.6 Hz, 1H), 7.70 (t, J = 7.6 Hz, 1H), 4.66 – 4.59 (m, 1H), 2.91 – 2.78 (m, 1H), 2.32 – 2.19 (m, 1H), 1.82 – 1.72 (m, 1H), 1.60 – 1.51 (m, 1H), 1.29 – 1.22 (m, 4H), 0.78 (t, J = 6.7 Hz, 3H).

^{13}C NMR (101 MHz, CDCl$_3$) δ 201.7, 151.7, 148.0, 143.9, 131.2, 130.9, 129.8, 127.8, 126.8 (q, J_{C-F} = 277.1 Hz), 124.2, 118.8, 38.0 (q, J_{C-F} = 2.3 Hz), 34.6 (q, J_{C-F} = 28.8 Hz), 32.3, 28.8, 22.4, 13.8.

^{19}F NMR (376 MHz, CDCl$_3$) δ -64.7.

HRMS (ESI-TOF): calcd for $[M+H]^+$ $C_{17}H_{18}ClF_3NO^+$ 344.1024; Found: 344.1023.

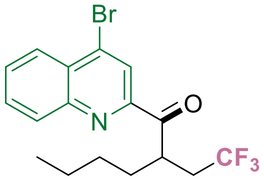

1-(4-bromoquinolin-2-yl)-2-(2,2,2-trifluoroethyl)hexan-1-one

The reaction solution was processed as general experimental procedure to afford the corresponding product **4d** as a colorless liquid (29.0 mg, 75%; PE/EA = 50/1).

^{1}H NMR (400 MHz, CDCl$_3$) δ 8.35 (s, 1H), 8.21 – 8.12 (m, 2H), 7.78 (t, J = 7.6 Hz, 1H), 7.70 (t, J = 7.6 Hz, 1H), 4.66 – 4.58 (m, 1H), 2.92 – 2.76 (m, 1H), 2.32 – 2.19 (m, 1H), 1.82 – 1.72 (m, 1H), 1.59 – 1.52 (m, 1H), 1.30 – 1.22 (m, 4H), 0.78 (t, J = 6.7 Hz,

3H).

^{13}C NMR (101 MHz, $CDCl_3$) δ 201.5, 151.4, 147.7, 135.3, 131.3, 130.9, 130.1, 129.2, 126.8, 126.7 (q, $J_{C\text{-}F}$ = 276.9 Hz), 122.7, 38.0 (q, $J_{C\text{-}F}$ = 2.1 Hz), 34.6 (q, $J_{C\text{-}F}$ = 28.3 Hz), 32.3, 28.8, 22.4, 13.8.

^{19}F NMR (376 MHz, $CDCl_3$) δ -64.7.

HRMS (ESI-TOF) m/z: $[M+H]^+$ Calcd. for $C_{17}H_{18}BrF_3NO^+$ 388.0518; Found: 388.0515.

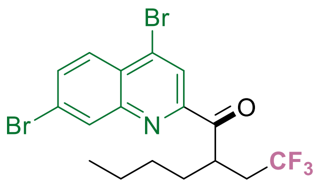

1-(4,7-dibromoquinolin-2-yl)-2-(2,2,2-trifluoroethyl)hexan-1-one

The reaction solution was processed as general experimental procedure to afford the corresponding product **4e** as a yellow liquid (32.6 mg, 70%; PE/EA = 50/1).

^{1}H NMR (400 MHz, $CDCl_3$) δ 8.42 (s, 2H), 8.12 (d, *J* = 9.0 Hz, 1H), 7.86 – 7.81 (m, 1H), 4.67 – 4.59 (m, 1H), 2.99 – 2.82 (m, 1H), 2.39 – 2.25 (m, 1H), 1.87 – 1.77 (m, 1H), 1.66 – 1.57 (m, 1H), 1.37 – 1.28 (m, 4H), 0.86 (t, *J* = 6.7 Hz, 3H).

^{13}C NMR (101 MHz, $CDCl_3$) δ 201.2, 152.2, 148.2, 135.4, 133.5, 133.2, 128.2, 128.0, 126.8 (q, $J_{C\text{-}F}$ = 276.3 Hz), 125.4, 123.0, 38.1 (q, $J_{C\text{-}F}$ = 2.0 Hz), 34.7 (q, $J_{C\text{-}F}$ = 28.5 Hz), 32.3, 28.8, 22.4, 13.8.

^{19}F NMR (376 MHz, $CDCl_3$) δ -64.8.

HRMS (ESI-TOF): calcd for $[M+H]^+$ $C_{17}H_{17}Br_2F_3NO^+$ 465.9624; Found: 465.9620.

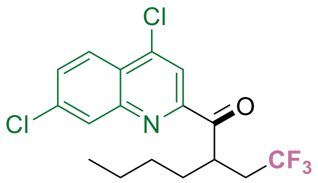

1-(4,7-dichloroquinolin-2-yl)-2-(2,2,2-trifluoroethyl)hexan-1-one

The reaction solution was processed as general experimental procedure to afford the corresponding product **4f** as a yellow liquid (24.5 mg, 65%; PE/EA = 50/1).

^{1}H NMR (400 MHz, $CDCl_3$) δ 8.30 – 8.16 (m, 3H), 7.74 – 7.69 (m, 1H), 4.68 – 4.60 (m, 1H), 2.98 – 2.83 (m, 1H), 2.39 – 2.26 (m, 1H), 1.88 – 1.77 (m, 1H), 1.67 – 1.57 (m, 1H), 1.38 – 1.28 (m, 4H), 0.86 (t, J = 6.7 Hz, 3H).

^{13}C NMR (101 MHz, $CDCl_3$) δ 201.4, 152.6, 148.4, 144.1, 137.2, 130.7, 129.9, 126.7 (q, $J_{C\text{-}F}$ = 276.7 Hz), 126.3, 125.6, 119.0, 38.0 (q, $J_{C\text{-}F}$ = 2.0 Hz), 34.7 (q, $J_{C\text{-}F}$ = 28.9 Hz), 32.3, 28.8, 22.4, 13.8.

^{19}F NMR (376 MHz, $CDCl_3$) δ -64.8.

HRMS (ESI-TOF): calcd for $[M+H]^+$ $C_{17}H_{17}Cl_2F_3NO^+$ 378.0634; Found: 378.0633.

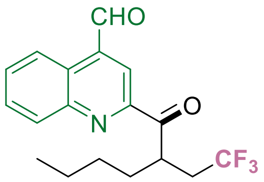

2-(2-(2,2,2-trifluoroethyl)hexanoyl)quinoline-4-carbaldehyde

The reaction solution was processed as general experimental procedure to afford the corresponding product **4g** as a colorless liquid (17.9 mg, 53%; PE/EA = 20/1).

^{1}H NMR (700 MHz, $CDCl_3$) δ 10.53 (s, 1H), 9.13 (d, *J* = 8.4 Hz, 1H), 8.56 (s, 1H), 8.34 (d, *J* = 8.3 Hz, 1H), 7.91 (t, *J* = 7.5 Hz, 1H), 7.86 (t, *J* = 7.6 Hz, 1H), 4.76 – 4.71 (m, 1H), 3.01 – 2.91 (m, 1H), 2.41 – 2.32 (m, 1H), 1.91 – 1.84 (m, 1H), 1.68 – 1.62 (m, 1H), 1.37 – 1.31 (m, 4H), 0.86 (t, *J* = 6.7 Hz, 3H).

^{13}C NMR (176 MHz, $CDCl_3$) δ 201.8, 192.9, 152.1, 148.3, 137.9, 131.8, 131.3, 130.8, 126.7 (q, $J_{C\text{-}F}$ = 276.3 Hz), 125.1, 124.9, 124.3, 37.9 (q, $J_{C\text{-}F}$ = 2.2 Hz), 34.6 (q, $J_{C\text{-}F}$ = 28.6 Hz), 32.3, 28.8, 22.4, 13.8.

^{19}F NMR (376 MHz, $CDCl_3$) δ -64.8.

HRMS (ESI-TOF): calcd for $[M+H]^+$ $C_{18}H_{19}F_3NO_2^+$ 338.1362; Found: 338.1368.

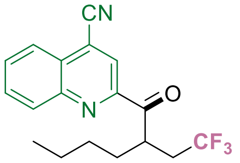

2-(2-(2,2,2-trifluoroethyl)hexanoyl)quinoline-4-carbonitrile

The reaction solution was processed as general experimental procedure to afford the corresponding product **4h** as a colorless liquid (16.7 mg, 50%; PE/EA = 50/1).

^{1}H NMR (700 MHz, CDCl$_3$) δ 8.48 (s, 1H), 8.34 (d, J = 8.4 Hz, 1H), 8.28 (d, J = 8.1 Hz, 1H), 7.96 (t, J = 7.6 Hz, 1H), 7.90 (t, J = 7.5 Hz, 1H), 4.70 – 4.65 (m, 1H), 2.96 – 2.89 (m, 1H), 2.38 – 2.31 (m, 1H), 1.87 – 1.81 (m, 1H), 1.65 – 1.60 (m, 1H), 1.36 – 1.29 (m, 4H), 0.86 (t, J = 6.4 Hz, 3H).

^{13}C NMR (176 MHz, CDCl$_3$) δ 201.0, 151.2, 147.0, 131.8, 131.6, 131.4, 127.1, 126.6 (q, $J_{C\text{-}F}$ = 276.8 Hz), 125.0, 122.9, 120.1, 115.2, 37.9 (q, $J_{C\text{-}F}$ = 2.1 Hz), 34.6 (q, $J_{C\text{-}F}$ = 28.7 Hz), 32.3, 28.8, 22.4, 13.8.

^{19}F NMR (376 MHz, CDCl$_3$) δ -64.8.

HRMS (ESI-TOF): calcd for $[M+H]^+$ $C_{18}H_{18}F_3N_2O^+$ 335.1366; Found: 335.1368.

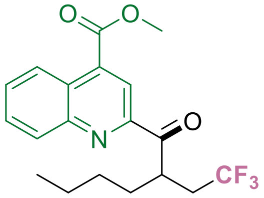

methyl 3-(2-(2,2,2-trifluoroethyl)hexanoyl)-1-naphthoate

The reaction solution was processed as general experimental procedure to afford the corresponding product **4i** as a colorless liquid (17.2 mg, 47%; PE/EA = 20/1).

^{1}H NMR (700 MHz, CDCl$_3$) δ 8.99 – 8.90 (m, 1H), 8.72 (s, 1H), 8.06 – 8.01 (m, 1H), 7.86 – 7.78 (m, 2H), 4.59 – 4.54 (m, 1H), 4.06 (s, 3H), 2.99 – 2.90 (m, 1H), 2.41 – 2.33 (m, 1H), 1.96 – 1.91 (m, 1H), 1.68 – 1.62 (m, 1H), 1.38 – 1.30 (m, 4H), 0.84 (t, J = 7.1 Hz, 3H).

^{13}C NMR (176 MHz, CDCl$_3$) δ 204.1, 165.7, 151.8, 139.9, 137.1, 131.4, 131.1, 128.6, 127.6, 127.4, 126.8, 126.8 (q, $J_{C\text{-}F}$ = 277.0 Hz), 52.8, 41.24 (q, $J_{C\text{-}F}$ = 1.8 Hz), 34.74 (q, $J_{C\text{-}F}$ = 28.4 Hz), 32.2, 28.8, 22.4, 13.8.

^{19}F NMR (376 MHz, CDCl$_3$) δ -64.3.

HRMS (ESI-TOF): calcd for $[M+H]^+$ $C_{19}H_{21}F_3NO_3^+$ 368.1468; Found: 368.1457.

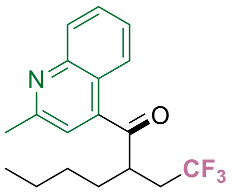

1-(2-methylquinolin-4-yl)-2-(2,2,2-trifluoroethyl)hexan-1-one

The reaction solution was processed as general experimental procedure to afford the corresponding product **4j** as a colorless liquid (19.1 mg, 59%; PE/EA = 50/1).

^{1}H NMR (700 MHz, CDCl$_3$) δ 8.15 (d, J = 8.4 Hz, 1H), 8.08 (d, J = 8.4 Hz, 1H), 7.74 (t, J = 7.6 Hz, 1H), 7.57 (t, J = 7.6 Hz, 1H), 7.42 (s, 1H), 3.66 – 3.62 (m, 1H), 3.07 – 2.98 (m, 1H), 2.83 (s, 3H), 2.35 – 2.27 (m, 1H), 1.76 – 1.71 (m, 1H), 1.56 – 1.51 (m, 1H), 1.31 – 1.20 (m, 4H), 0.80 (t, J = 7.1 Hz, 3H).

^{13}C NMR (176 MHz, CDCl$_3$) δ 203.7, 158.4, 148.8, 143.3, 130.1, 129.3, 127.5, 126.9 (q, $J_{C\text{-}F}$ = 276.2 Hz), 124.9, 122.4, 119.9, 44.1 (q, $J_{C\text{-}F}$ = 2.0 Hz), 33.9 (q, $J_{C\text{-}F}$ = 28.6 Hz), 31.8, 28.6, 25.5, 22.4, 13.7.

^{19}F NMR (376 MHz, CDCl$_3$) δ -64.7.

HRMS (ESI-TOF): calcd for $[M+H]^+$ $C_{18}H_{21}F_3NO_2^+$ 324.1570; Found: 324.1566.

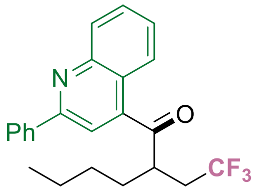

1-(2-phenylquinolin-4-yl)-2-(2,2,2-trifluoroethyl)hexan-1-one

The reaction solution was processed as general experimental procedure to afford the corresponding product **4k** as a yellow liquid (27.7 mg, 72%; PE/EA = 50/1).

^{1}H NMR (700 MHz, CDCl$_3$) δ 8.26 – 8.19 (m, 2H), 8.20 – 8.14 (m, 2H), 8.02 (s, 1H), 7.81 – 7.77 (m, 1H), 7.63 – 7.60 (m, 1H), 7.60 – 7.55 (m, 2H), 7.54 – 7.50 (m, 1H), 3.76 – 3.72 (m, 1H), 3.10 – 3.02 (m, 1H), 2.38 – 2.31 (m, 1H), 1.82 – 1.76 (m, 1H), 1.61 – 1.56 (m, 1H), 1.36 – 1.24 (m, 4H), 0.80 (t, J = 7.2 Hz, 3H).

^{13}C NMR (176 MHz, CDCl$_3$) δ 203.9, 156.8, 149.2, 144.0, 138.8, 130.4, 130.3, 129.9, 129.1, 128.1, 127.5, 126.7 (q, $J_{C\text{-}F}$ = 277.0 Hz), 124.9, 123.0, 117.0, 44.2 (q, $J_{C\text{-}F}$ = 1.9 Hz), 34.2 (q, $J_{C\text{-}F}$ = 28.6 Hz), 32.0, 28.7, 22.4, 13.7.

HRMS (ESI-TOF): calcd for $[M+H]^+$ $C_{23}H_{23}F_3NO^+$ 386.1726; Found: 386.1726.

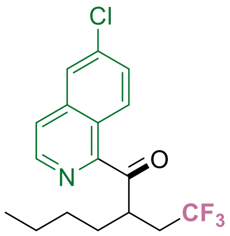

1-(4-chloroquinolin-2-yl)-2-(2,2,2-trifluoroethyl)hexan-1-one

The reaction solution was processed as general experimental procedure to afford the corresponding product **4l** as a yellow liquid (19.9 mg, 58%; PE/EA = 50/1).

^{1}H NMR (700 MHz, CDCl$_3$) δ 8.88 (d, J = 9.2 Hz, 1H), 8.63 (d, J = 5.5 Hz, 1H), 7.87 (s, 1H), 7.75 (d, J = 5.5 Hz, 1H), 7.62 (dd, J = 9.2, 1.6 Hz, 1H), 4.67 – 4.34 (m, 1H), 3.03 – 2.78 (m, 1H), 2.40 – 2.18 (m, 1H), 1.91 – 1.69 (m, 1H), 1.61 – 1.57 (m, 1H), 1.38 – 1.20 (m, 4H), 0.82 (t, J = 6.9 Hz, 3H).

^{13}C NMR (176 MHz, CDCl$_3$) δ 204.3, 151.9, 142.3, 137.9, 136.8, 130.3, 128.5, 126.8 (q, J_{C-F} = 277.0 Hz), 125.8, 124.5, 123.8, 40.5 (q, J_{C-F} = 1.9 Hz), 34.5 (q, J_{C-F} = 28.5 Hz), 32.1, 28.8, 22.5, 13.8.

^{19}F NMR (376 MHz, CDCl$_3$) δ -64.6.

HRMS (ESI-TOF): calcd for $[M+H]^+$ $C_{17}H_{18}ClF_3NO^+$ 344.1024; Found: 344.1018.

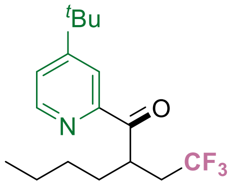

1-(4-(tert-butyl)pyridin-2-yl)-2-(2,2,2-trifluoroethyl)hexan-1-one

The reaction solution was processed as general experimental procedure to afford the corresponding product **4m** as a colorless liquid (17.3 mg, 55%; PE/EA = 50/1).

^{1}H NMR (700 MHz, CDCl$_3$) δ 8.60 (d, J = 5.1 Hz, 1H), 8.09 (d, J = 2.0 Hz, 1H), 7.48 (dd, J = 5.1, 2.0 Hz, 1H), 4.53 – 4.49 (m, 1H), 2.91 – 2.83 (m, 1H), 2.31 – 2.23 (m, 1H), 1.80 – 1.74 (m, 1H), 1.56 – 1.52 (m, 1H), 1.35 (s, 9H), 1.31 – 1.26 (m, 4H), 0.86 – 0.83 (m, 3H).

^{13}C NMR (176 MHz, CDCl$_3$) δ 202.7, 161.3, 152.3, 149.0, 126.8 (q, J_{C-F} = 277.0 Hz), 124.5, 119.5, 38.2 (q, J_{C-F} = 2.2 Hz), 35.0, 34.6 (q, J_{C-F} = 28.4 Hz), 32.1, 30.5, 28.7, 22.5, 13.8.

^{19}F NMR (376 MHz, $CDCl_3$) δ -64.7.

HRMS (ESI-TOF): calcd for $[M+H]^+$ $C_{17}H_{25}F_3NO^+$ 316.1883; Found: 316.1869.

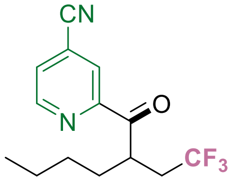

2-(2-(2,2,2-trifluoroethyl)hexanoyl)isonicotinonitrile

The reaction solution was processed as general experimental procedure to afford the corresponding product **4n** as a colorless liquid (14.8 mg, 52%; PE/EA = 50/1).

^{1}H NMR (700 MHz, $CDCl_3$) δ 8.90 (d, J = 4.9 Hz, 1H), 8.28 (s, 1H), 7.72 (dd, J = 4.9, 1.2 Hz, 1H), 4.45 – 4.41 (m, 1H), 2.91 – 2.83 (m, 1H), 2.33 – 2.26 (m, 1H), 1.78 – 1.73 (m, 1H), 1.34 – 1.25 (m, 5H), 0.85 (t, J = 7.0 Hz, 3H).

^{13}C NMR (176 MHz, $CDCl_3$) δ 200.6, 153.2, 150.1, 128.4, 126.6 (q, J_{C-F} = 277.0 Hz), 124.3, 122.0, 115.8, 38.2 (q, J_{C-F} = 2.0 Hz), 34.5 (q, J_{C-F} = 28.6 Hz), 32.1, 28.7, 22.5, 13.7.

^{19}F NMR (376 MHz, $CDCl_3$) δ -64.9.

HRMS (ESI-TOF): calcd for $[M+H]^+$ $C_{14}H_{16}F_3N_2O^+$ 285.1209; Found: 285.1199.

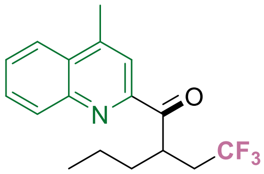

1-(4-methylquinolin-2-yl)-2-(2,2,2-trifluoroethyl)pentan-1-one

The reaction solution was processed as general experimental procedure to afford the corresponding product **4q** as a yellow liquid (13.3 mg, 43%; PE/EA = 50/1).

^{1}H NMR (700 MHz, $CDCl_3$) δ 8.27 – 8.17 (m, 1H), 8.04 (d, J = 8.3 Hz, 1H), 7.98 (s, 1H), 7.78 (t, J = 7.5 Hz, 1H), 7.68 (t, J = 7.5 Hz, 1H), 4.93 – 4.59 (m, 1H), 2.97 – 2.86 (m, 1H), 2.77 (s, 3H), 2.36 – 2.24 (m, 1H), 1.86 – 1.75 (m, 1H), 1.67 – 1.57 (m, 1H), 1.41 – 1.29 (m, 2H), 0.92 (t, J = 7.3 Hz, 3H).

^{13}C NMR (176 MHz, $CDCl_3$) δ 203.1, 151.5, 147.1, 145.5, 131.5, 129.7, 129.6, 128.5, 126.9 (q, J_{C-F} = 276.9 Hz), 123.8, 119.1, 37.7 (q, J_{C-F} = 2.1 Hz), 34.9, 34.7 (q, J_{C-F} = 28.4 Hz), 20.0, 18.9, 13.9.

^{19}F NMR (376 MHz, CDCl$_3$) δ -64.7.

HRMS (ESI-TOF): calcd for $[M+H]^+$ $C_{17}H_{19}F_3NO^+$ 310.1413, found: 310.1408.

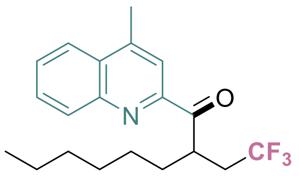

1-(4-methylquinolin-2-yl)-2-(2,2,2-trifluoroethyl)octan-1-one

The reaction solution was processed as general experimental procedure to afford the corresponding product **4r** as a yellow liquid (14.4 mg, 41%; PE/EA = 50/1).

^{1}H NMR (700 MHz, CDCl$_3$) δ 8.21 (d, J = 8.4 Hz, 1H), 8.04 (d, J = 8.3 Hz, 1H), 7.98 (s, 1H), 7.78 (t, J = 7.5 Hz, 1H), 7.68 (t, J = 7.5 Hz, 1H), 4.95 – 4.58 (m, 1H), 2.97 – 2.86 (m, 1H), 2.77 (s, 3H), 2.35 – 2.29 (m, 1H), 1.90 – 1.78 (m, 1H), 1.67 – 1.58 (m, 1H), 1.34 – 1.20 (m, 8H), 0.83 (t, J = 7.1 Hz, 3H).

^{13}C NMR (176 MHz, CDCl$_3$) δ 203.1, 151.5, 147.1, 145.5, 131.5, 129.7, 129.6, 128.5, 126.9 (q, $J_{C\text{-}F}$ = 276.8 Hz), 123.8, 119.1, 37.9 (q, $J_{C\text{-}F}$ = 1.9 Hz), 34.6 (q, $J_{C\text{-}F}$ = 28.5 Hz), 32.6, 31.5, 29.0, 26.6, 22.6, 18.9, 14.0.

^{19}F NMR (376 MHz, CDCl$_3$) δ -64.7.

HRMS (ESI-TOF): calcd for $[M+H]^+$ $C_{20}H_{25}F_3NO^+$ 352.1883 found: 352.1880.

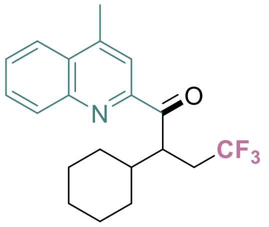

2-cyclohexyl-4,4,4-trifluoro-1-(4-methylquinolin-2-yl)butan-1-one

The reaction solution was processed as general experimental procedure to afford the corresponding product **4s** as a white solid (19.5 mg, 56%; PE/EA = 50/1).

^{1}H NMR (700 MHz, CDCl$_3$) δ 8.23 (d, J = 8.4 Hz, 1H), 8.04 (d, J = 8.3 Hz, 1H), 7.99 (s, 1H), 7.77 (t, J = 7.5 Hz, 1H), 7.67 (t, J = 7.5 Hz, 1H), 4.72 (dd, J = 10.3, 5.2 Hz, 1H), 2.97 – 2.90 (m, 1H), 2.76 (s, 3H), 2.39 – 2.25 (m, 1H), 1.88 – 1.58 (m, 7H), 1.23 – 1.16 (m, 2H), 1.10 (dd, J = 25.3, 12.6 Hz, 2H).

^{13}C NMR (101 MHz, CDCl$_3$) δ 202.9, 152.0, 147.0, 145.4, 131.6, 129.7, 129.5, 128.4, 127.2 (d, J_{C-F} = 276.9 Hz), 123.8, 119.0, 42.7, 40.5, 32.2 (q, J_{C-F} = 28.4 Hz), 31.0, 29.3, 26.4, 26.3, 26.2, 18.9.

^{19}F NMR (376 MHz, CDCl$_3$) δ -65.0.

HRMS (ESI-TOF): calcd for $[M+H]^+$ $C_{20}H_{23}F_3NO^+$ 350.1726, found: 350.1720.

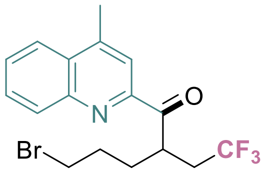

5-bromo-1-(4-methylquinolin-2-yl)-2-(2,2,2-trifluoroethyl)pentan-1-one

The reaction solution was processed as general experimental procedure to afford the corresponding product **4t** as a yellow liquid (15.5 mg, 40%; PE/EA = 50/1).

^{1}H NMR (700 MHz, CDCl$_3$) δ 8.22 (d, J = 8.4 Hz, 1H), 8.05 (d, J = 8.3 Hz, 1H), 7.98 (s, 1H), 7.79 (t, J = 7.5 Hz, 1H), 7.69 (t, J = 7.6 Hz, 1H), 5.09 – 4.53 (m, 1H), 3.47 – 3.38 (m, 2H), 2.95 – 2.88 (m, 1H), 2.78 (s, 3H), 2.38 – 2.27 (m, 1H), 2.04 – 1.96 (m, 1H), 1.94 – 1.81 (m, 3H).

^{13}C NMR (176 MHz, CDCl$_3$) δ 202.5, 151.3, 147.0, 145.8, 131.5, 129.8, 129.8, 128.7, 126.6 (d, J_{C-F} = 277.0 Hz), 123.8, 119.0, 37.1 (d, J_{C-F} = 1.9 Hz), 35.2 (q, J_{C-F} = 28.6 Hz), 32.9, 31.3, 29.9, 18.9.

^{19}F NMR (376 MHz, CDCl$_3$) δ -64.6.

HRMS (ESI-TOF): calcd for $[M+H]^+$ $C_{17}H_{18}BrF_3NO^+$ 388.0518, found: 388.0513.

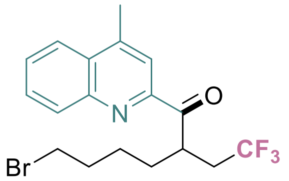

6-bromo-1-(4-methylquinolin-2-yl)-2-(2,2,2-trifluoroethyl)hexan-1-one

The reaction solution was processed as general experimental procedure to afford the corresponding product **4u** as a yellow liquid (18.8 mg, 47%; PE/EA = 50/1).

^{1}H NMR (700 MHz, CDCl$_3$) δ 8.21 (d, J = 8.4 Hz, 1H), 8.05 (d, J = 8.3 Hz, 1H), 7.99 (s, 1H), 7.79 (t, J = 7.6 Hz, 1H), 7.69 (t, J = 7.6 Hz, 1H), 4.84 – 4.62 (m, 1H), 3.43 –

3.27 (m, 2H), 3.04 – 2.85 (m, 1H), 2.77 (s, 3H), 2.46 – 2.24 (m, 1H), 1.99 – 1.82 (m, 3H), 1.78 – 1.61 (m, 1H), 1.54 – 1.43 (m, 2H).

^{13}C NMR (176 MHz, CDCl$_3$) δ 202.6, 151.3, 147.0, 145.7, 131.4, 129.8, 129.8, 128.6, 126.8 (q, J_{C-F} = 276.9 Hz), 123.8, 119.1, 37.6 (q, J_{C-F} = 1.8 Hz), 34.7 (q, J_{C-F} = 28.6 Hz), 33.2, 32.2, 31.6, 25.2, 18.9.

^{19}F NMR (376 MHz, CDCl$_3$) δ -64.6.

HRMS (ESI-TOF): calcd for $[M+H]^+$ $C_{18}H_{20}BrF_3NO^+$ 402.0675, found: 402.0667.

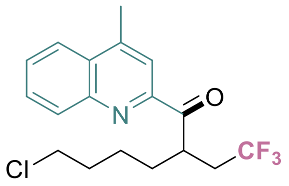

6-chloro-1-(4-methylquinolin-2-yl)-2-(2,2,2-trifluoroethyl)hexan-1-one

The reaction solution was processed as general experimental procedure to afford the corresponding product **4v** as a yellow liquid (15.0 mg, 42%; PE/EA = 50/1).

^{1}H NMR (700 MHz, CDCl$_3$) δ 8.21 (d, J = 8.4 Hz, 1H), 8.05 (d, J = 8.3 Hz, 1H), 7.99 (s, 1H), 7.79 (t, J = 7.6 Hz, 1H), 7.69 (t, J = 7.6 Hz, 1H), 4.88 – 4.55 (m, 1H), 3.52 – 3.46 (m, 2H), 3.01 – 2.87 (m, 1H), 2.77 (s, 3H), 2.37 – 2.28 (m, 1H), 1.93 – 1.76 (m, 3H), 1.73 – 1.61 (m, 1H), 1.52 – 1.47 (m, 2H).

^{13}C NMR (176 MHz, CDCl$_3$) δ 202.7, 151.3, 147.0, 145.7, 131.4, 129.8, 129.8, 128.6, 126.8 (q, J_{C-F} = 276.9 Hz), 123.8, 119.1, 44.5, 37.6 (q, J_{C-F} = 1.9 Hz), 34.7 (q, J_{C-F} = 28.5 Hz), 32.1, 31.7, 24.0, 18.9.

^{19}F NMR (376 MHz, CDCl$_3$) δ -64.6.

HRMS (ESI-TOF): calcd for $[M+H]^+$ $C_{18}H_{20}ClF_3NO^+$ 358.1180, found: 358.1179.

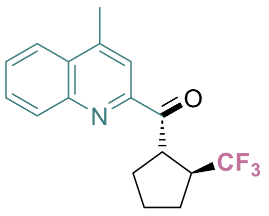

(4-methylquinolin-2-yl)(2-(trifluoromethyl)cyclopentyl)methanone

The reaction solution was processed as general experimental procedure to afford the corresponding product **4w** as a white solid (13.2 mg, 43%; PE/EA = 50/1).

^{1}H NMR (400 MHz, CDCl$_3$) δ 8.22 (d, J = 8.4 Hz, 1H), 8.04 (d, J = 8.3 Hz, 1H), 7.99 (s, 1H), 7.78 (t, J = 7.5 Hz, 1H), 7.67 (t, J = 7.5 Hz, 1H), 4.78 (dd, J = 16.5, 7.3 Hz, 1H), 3.54 – 3.35 (m, 1H), 2.77 (s, 3H), 2.48 – 2.38 (m, 1H), 2.13 – 2.04 (m, 1H), 1.97 – 1.91 (m, 1H), 1.90 – 1.83 (m, 1H), 1.79 – 1.67 (m, 2H).

^{13}C NMR (176 MHz, CDCl$_3$) δ 201.8, 151.6, 147.1, 145.4, 131.4, 129.7, 129.6, 128.5, 128.4 (q, J_{C-F} = 277.5 Hz), 123.8, 119.2, 45.8 (q, J_{C-F} = 1.7 Hz), 43.9 (q, J_{C-F} = 27.2 Hz), 33.0, 27.1, 25.9, 18.9.

^{19}F NMR (376 MHz, CDCl$_3$) δ -70.3.

HRMS (ESI-TOF): calcd for $[M+H]^+$ $C_{17}H_{17}F_3NO^+$ 308.1257:, found: 308.1258.

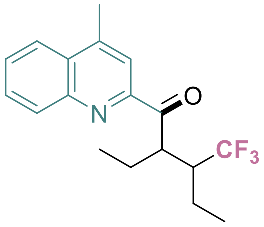

2-ethyl-1-(4-methylquinolin-2-yl)-3-(trifluoromethyl)pentan-1-one

The reaction solution was processed as general experimental procedure to afford the corresponding product **4x** as a yellow liquid (14.2 mg, 44%, dr = 2:3; PE/EA = 50/1).

^{1}H NMR (700 MHz, CDCl$_3$) δ 8.20 (dd, J = 21.5, 8.4 Hz, 1H), 8.08 – 7.94 (m, 2H), 7.77 (t, J = 7.6 Hz, 1H), 7.68 (t, J = 7.5 Hz, 1H), 4.74 – 4.64 (m, 0.4H), 4.62 – 4.53 (m, 0.6H), 2.77 (s, 3H), 2.08 – 1.90 (m, 1H), 1.82 – 1.54 (m, 4H), 1.19 – 0.96 (m, 3H), 0.93 – 0.82 (m, 3H).

^{13}C NMR (176 MHz, CDCl$_3$) δ 203.3, 203.0, 152.3, 152.0, 147.0, 146.9, 145.7, 145.5, 131.5, 131.2, 129.7, 129.6, 128.5, 128.4, 128.4 (q, J_{C-F} = 281.7 Hz), 128.3 (q, J_{C-F} = 281.8 Hz), 123.8, 123.7, 119.1, 119.0, 45.8 (q, J_{C-F} = 24.5 Hz), 45.4 (q, J_{C-F} = 24.1 Hz), 44.4, 43.5 (q, J_{C-F} = 1.7 Hz), 20.5, 20.4, 18.9, 18.1, 18.1, 12.4, 12.1, 12.0, 11.9.

^{19}F NMR (376 MHz, CDCl$_3$) δ-65.2, -66.4.

HRMS (ESI-TOF): calcd for $[M+H]^+$ $C_{18}H_{21}F_3NO^+$ 324.1570, found: 324.1565.

7. The NMR spectrum

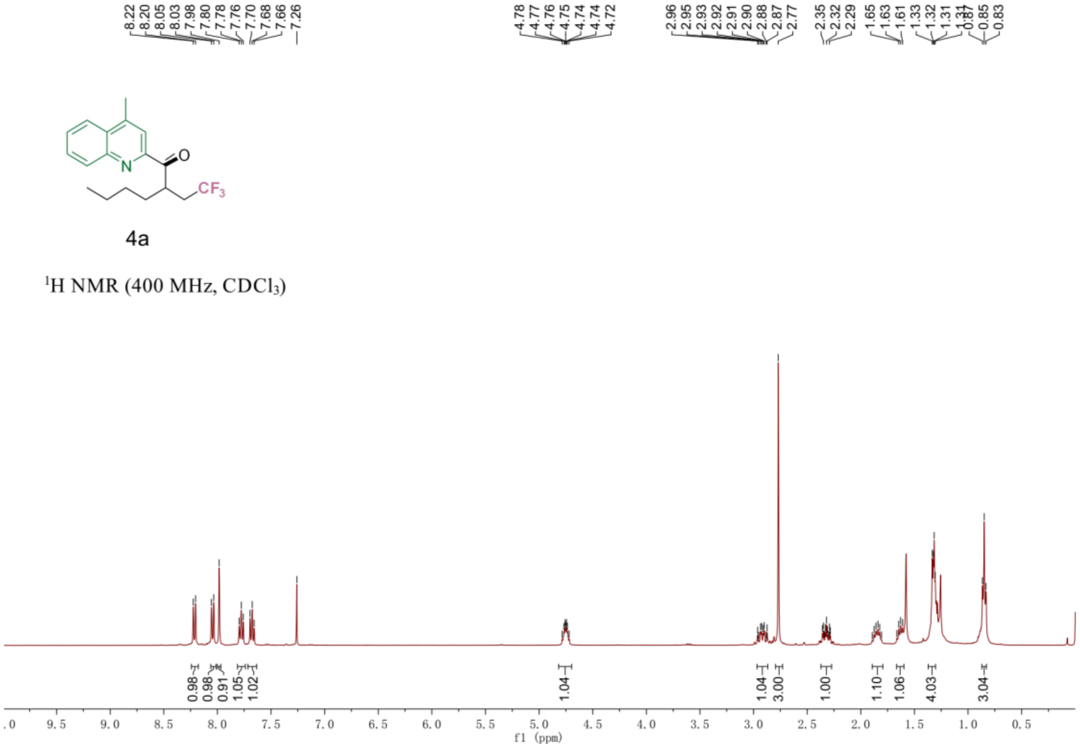

^{1}H NMR (400 MHz, $CDCl_3$)

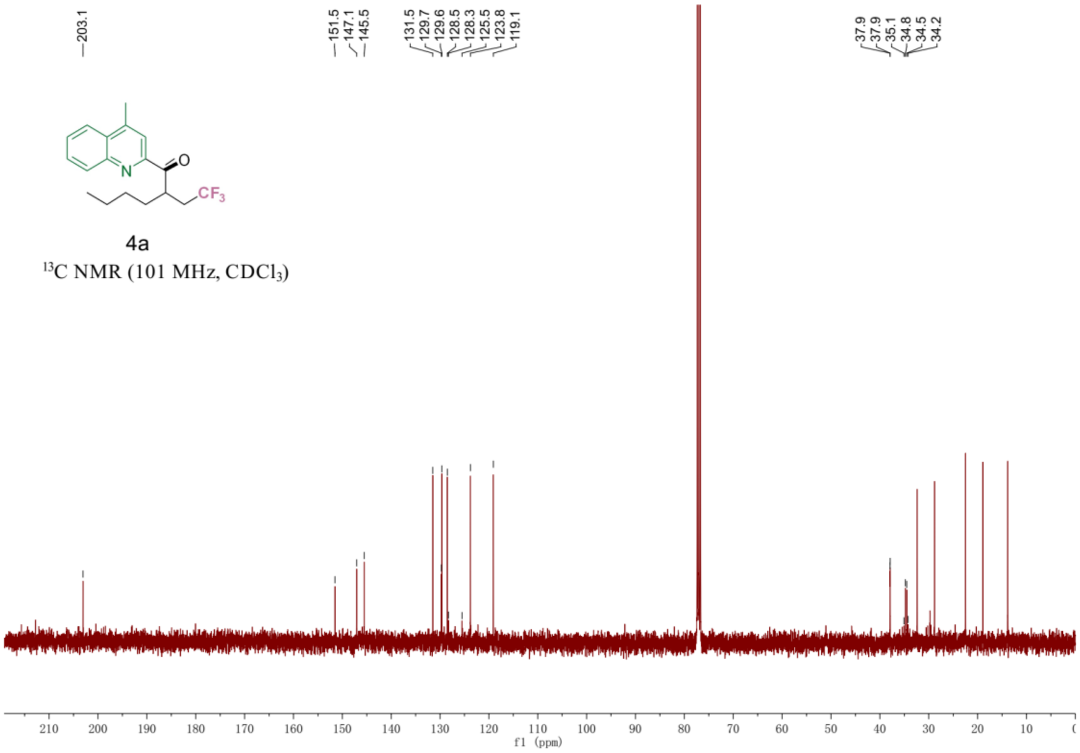

^{13}C NMR (101 MHz, $CDCl_3$)

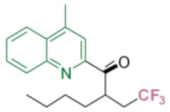

4a

^{19}F NMR (376 MHz, $CDCl_3$)

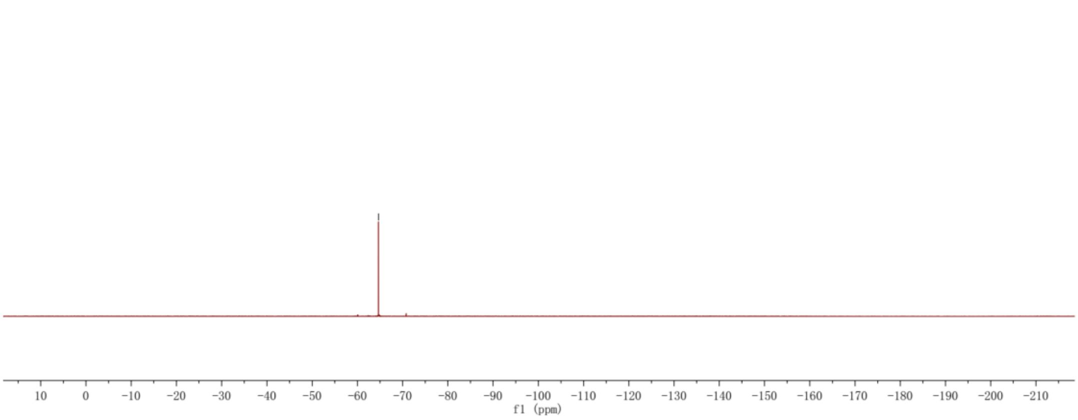

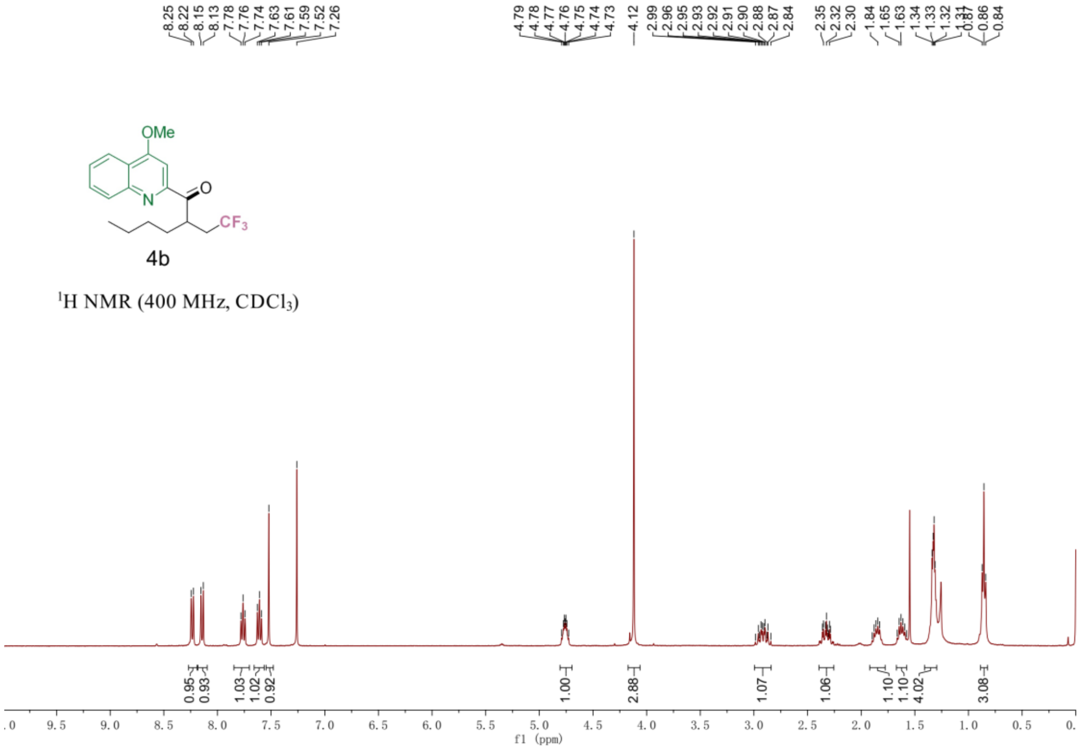
OMe
N
O
CF3
4b
1H NMR (400 MHz, CDCl3)
f1 (ppm)

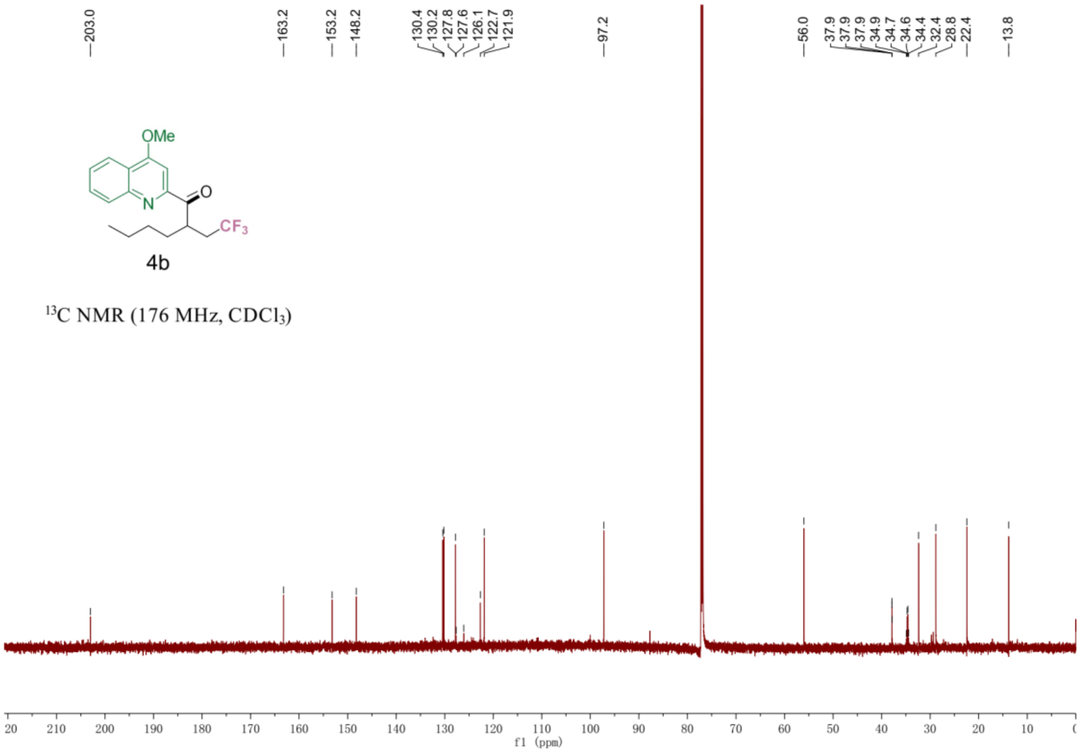
OMe
N
O
CF3
4b
13C NMR (176 MHz, CDCl3)
f1 (ppm)

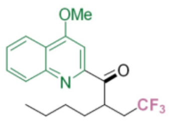

4b

^{19}F NMR (376 MHz, $CDCl_3$)

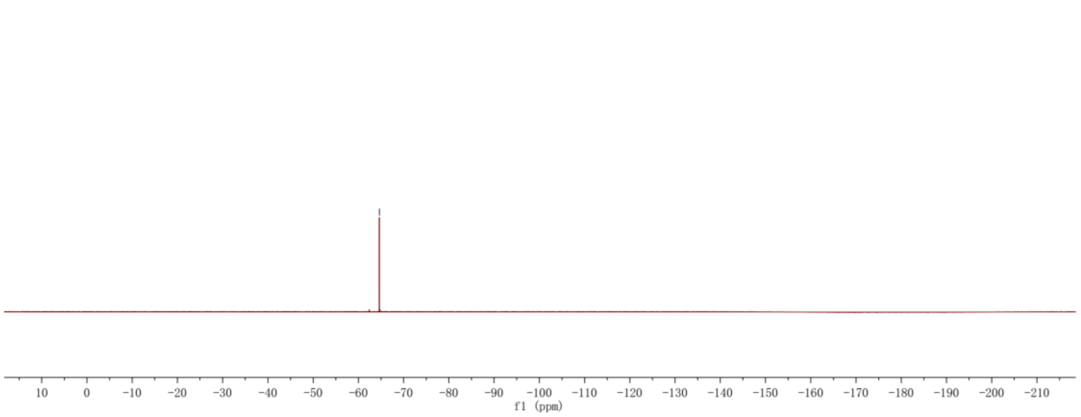

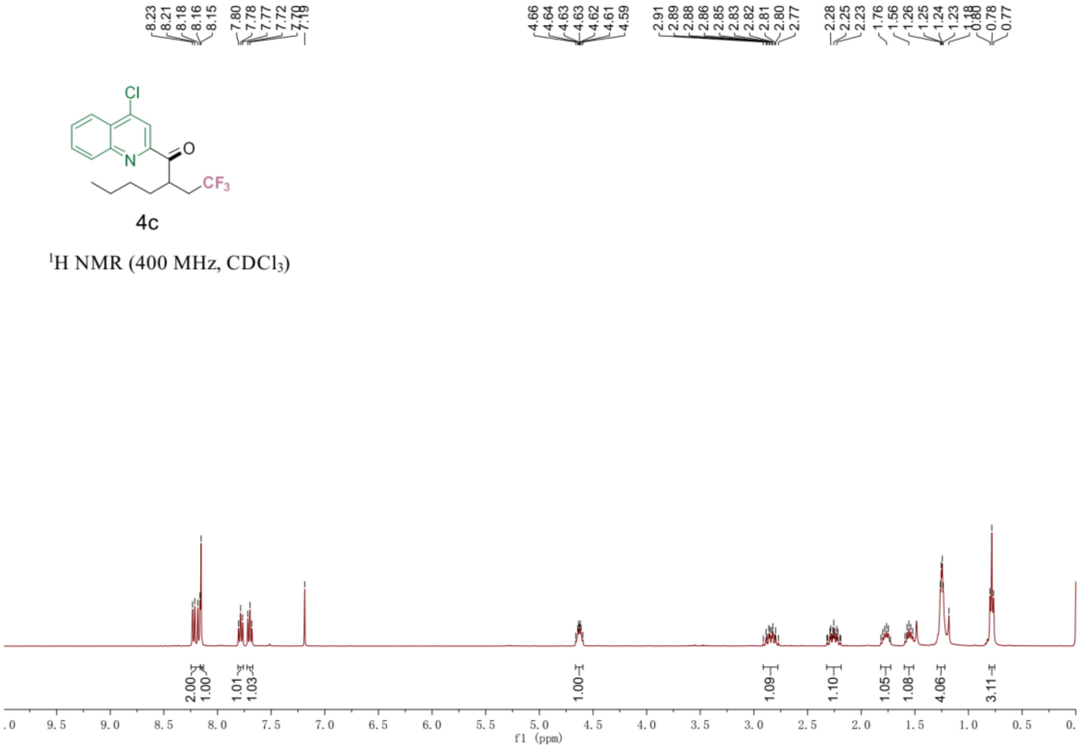

4c

^{1}H NMR (400 MHz, $CDCl_3$)

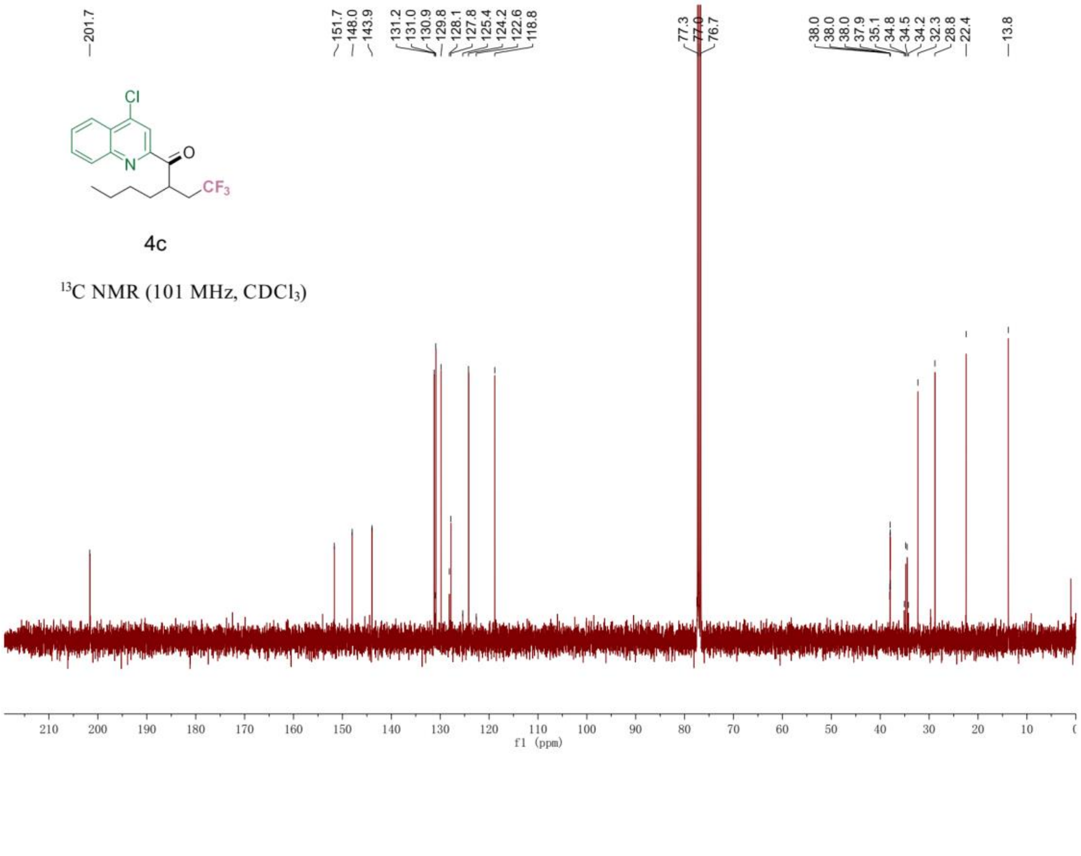

4c

^{13}C NMR (101 MHz, $CDCl_3$)

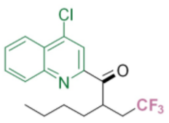

4c

^{19}F NMR (376 MHz, $CDCl_3$)

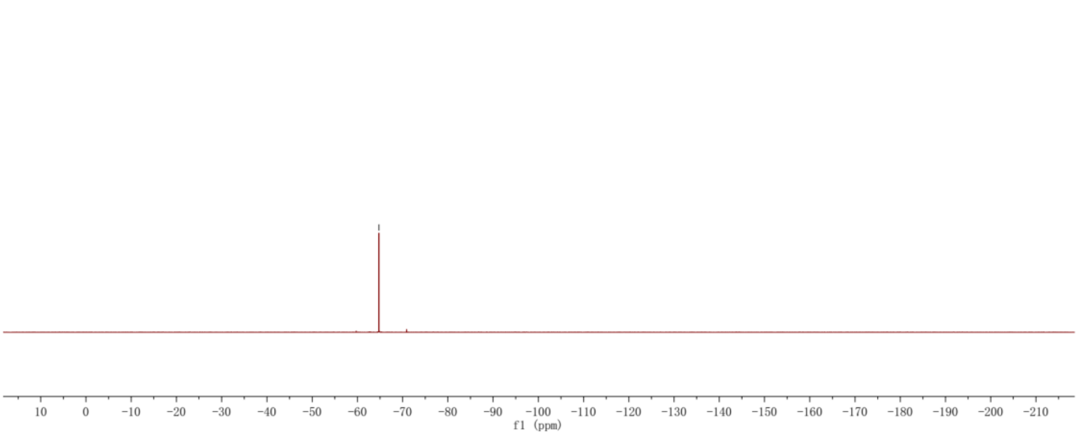

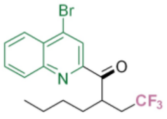

4d

^{1}H NMR (400 MHz, $CDCl_3$)

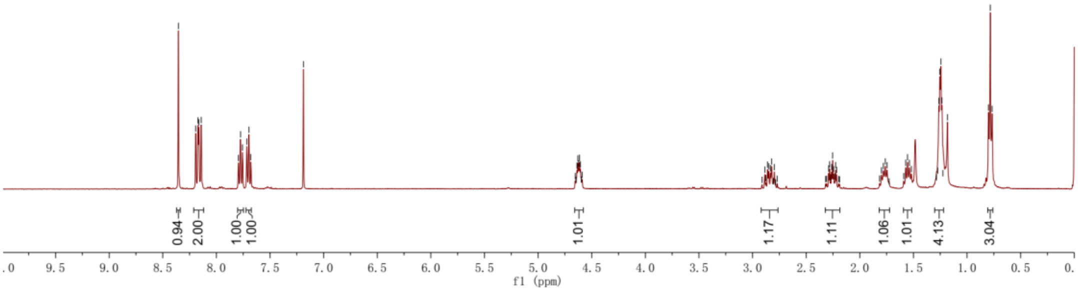

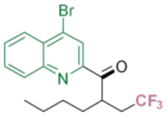

4d

^{13}C NMR (101 MHz, $CDCl_3$)

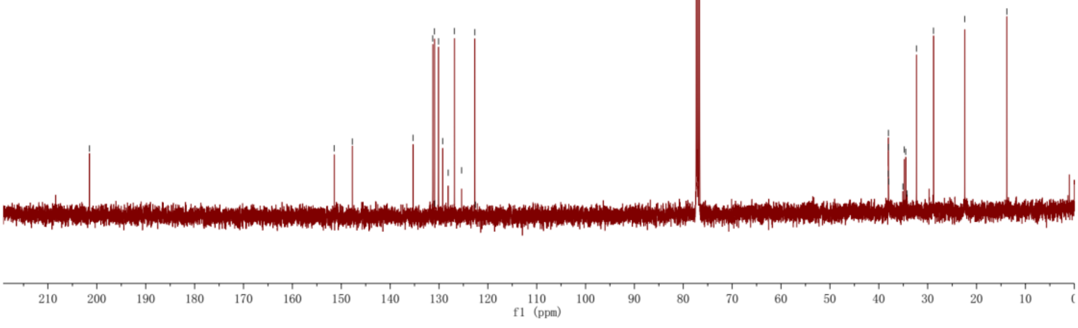

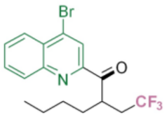

4d

^{19}F NMR (376 MHz, $CDCl_3$)

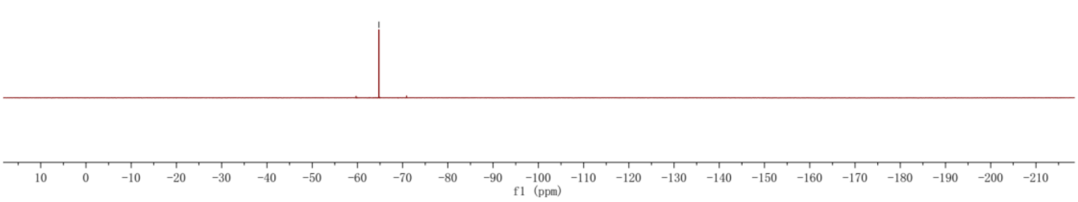

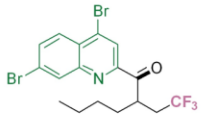

4e

^{1}H NMR (400 MHz, $CDCl_3$)

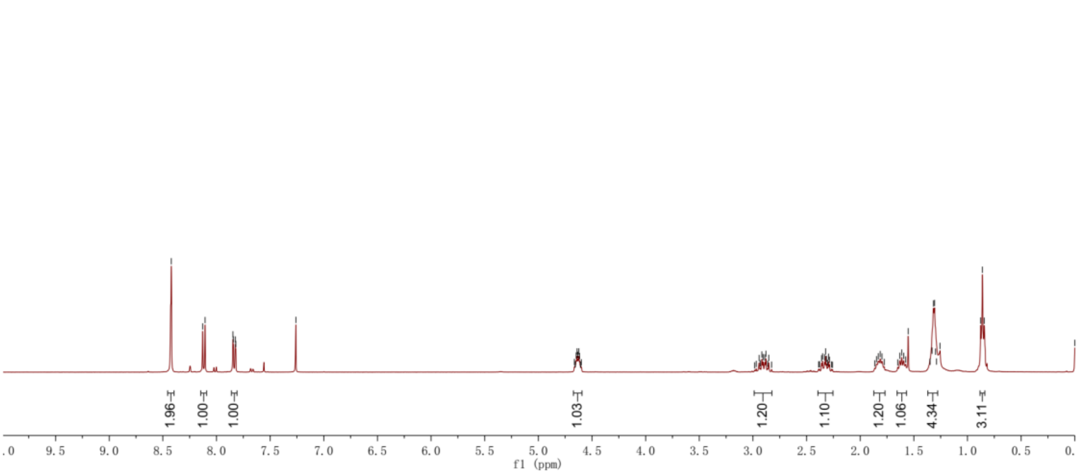

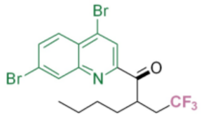

4e

^{13}C NMR (176 MHz, $CDCl_3$)

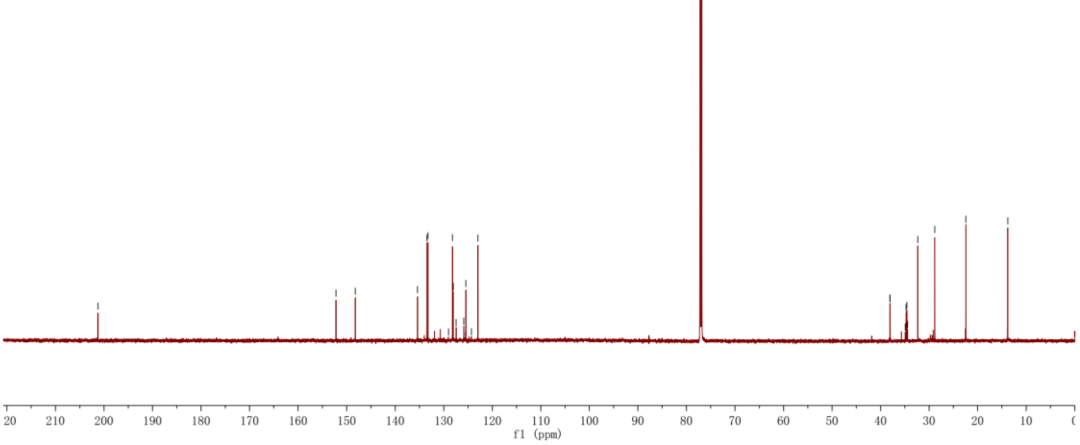

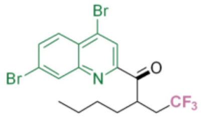

4e

^{19}F NMR (376 MHz, $CDCl_3$)

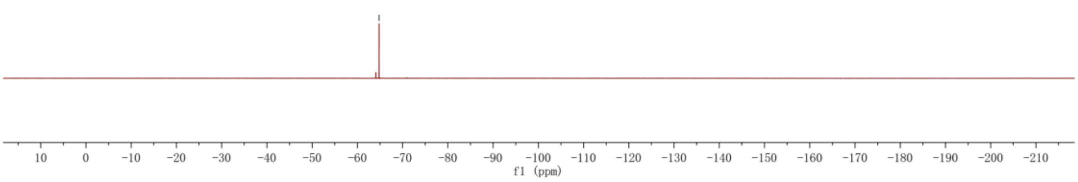

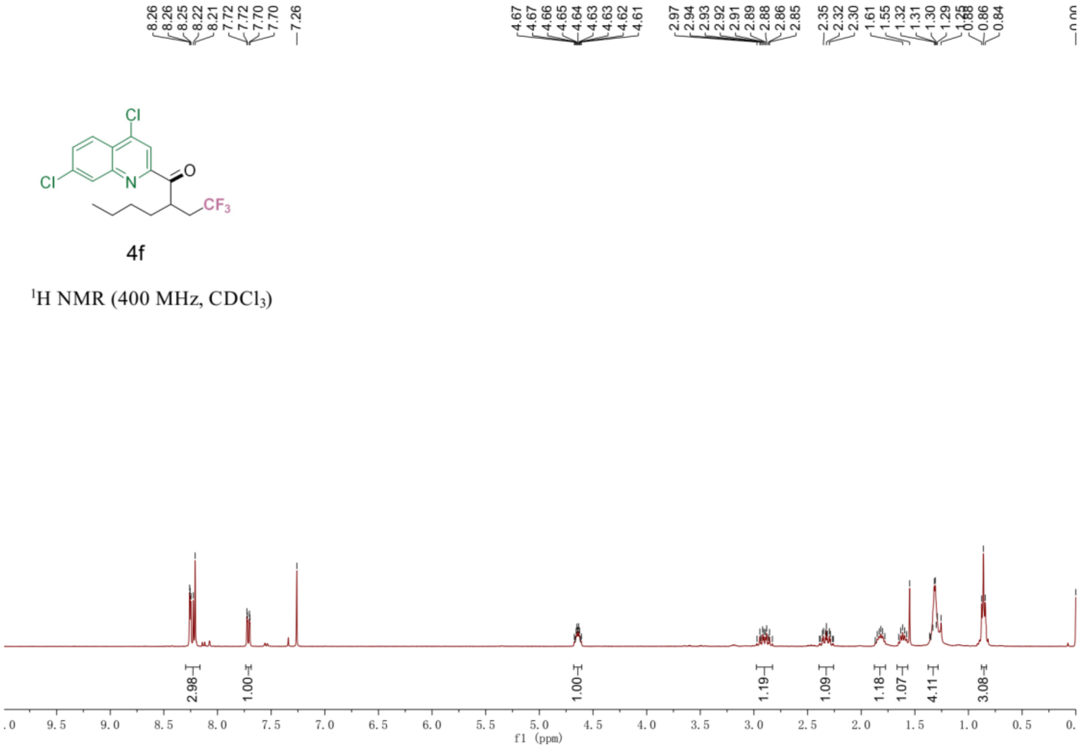

4f

^{1}H NMR (400 MHz, $CDCl_3$)

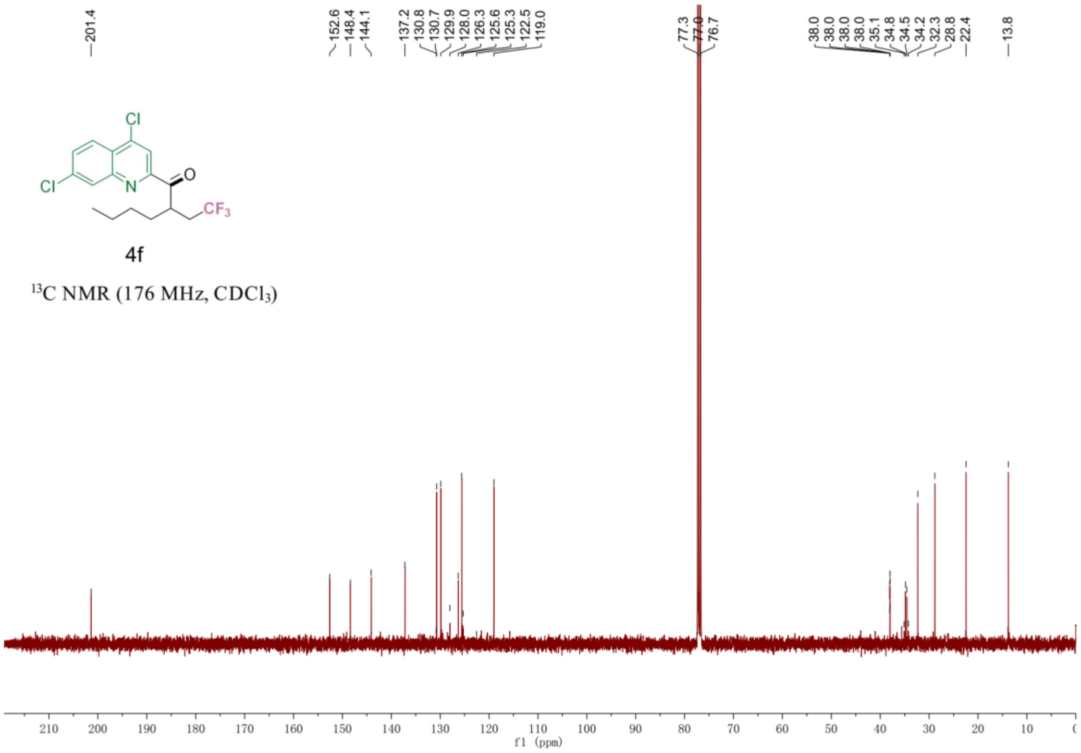

4f

^{13}C NMR (176 MHz, $CDCl_3$)

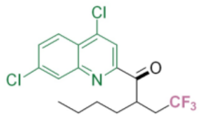

4f

^{19}F NMR (376 MHz, $CDCl_3$)

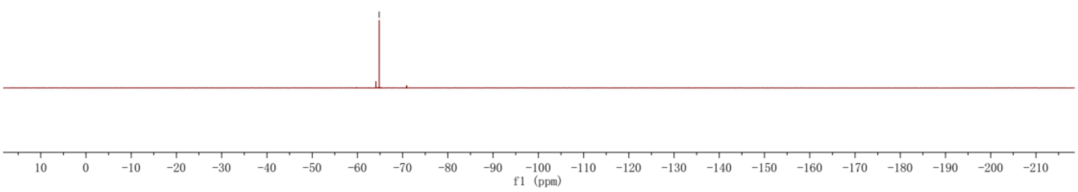

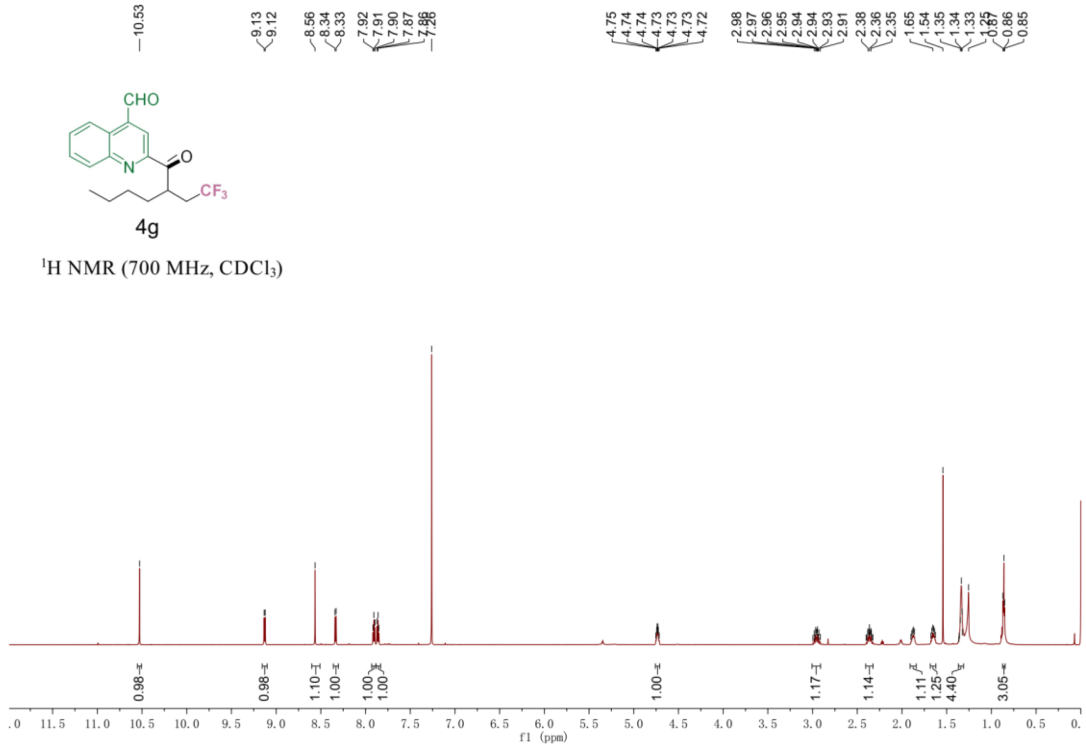

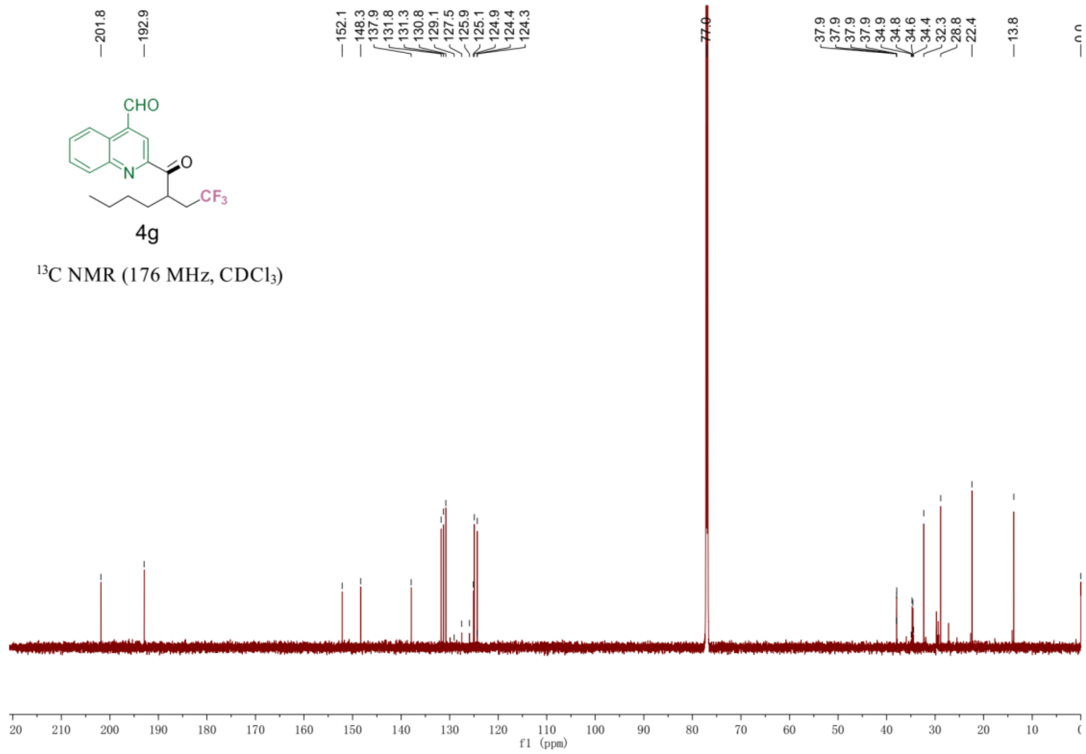

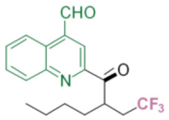

4g

^{19}F NMR (376 MHz, $CDCl_3$)

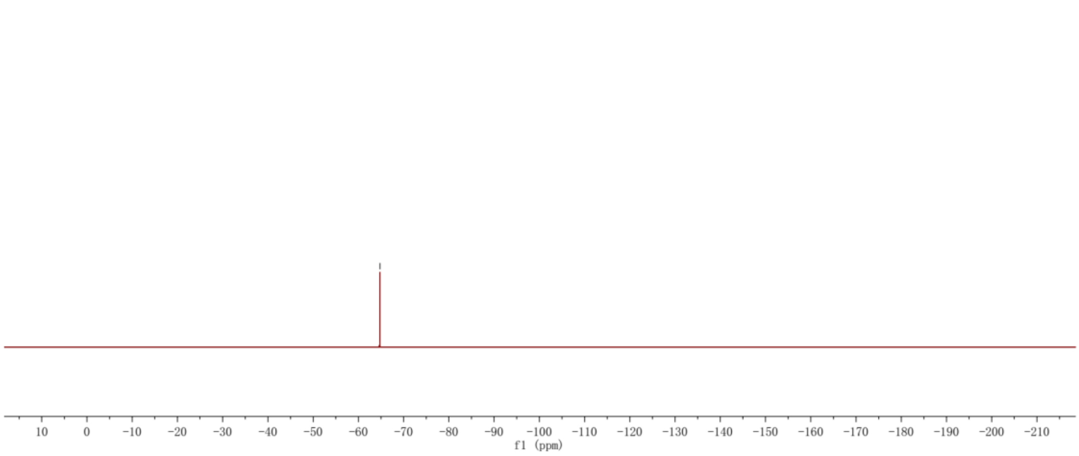

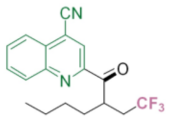

4h

^{1}H NMR (700 MHz, $CDCl_3$)

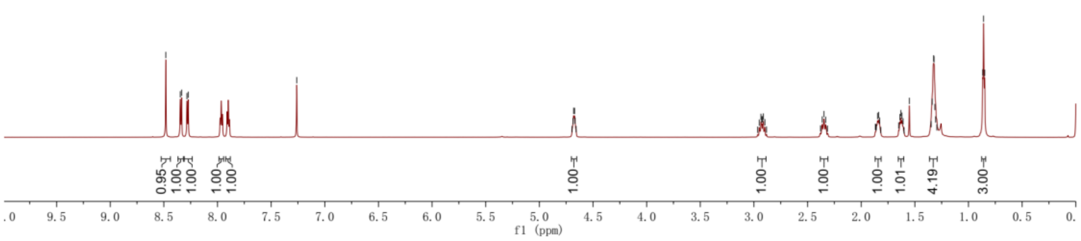

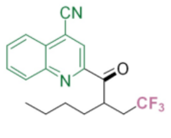

4h

^{13}C NMR (176 MHz, $CDCl_3$)

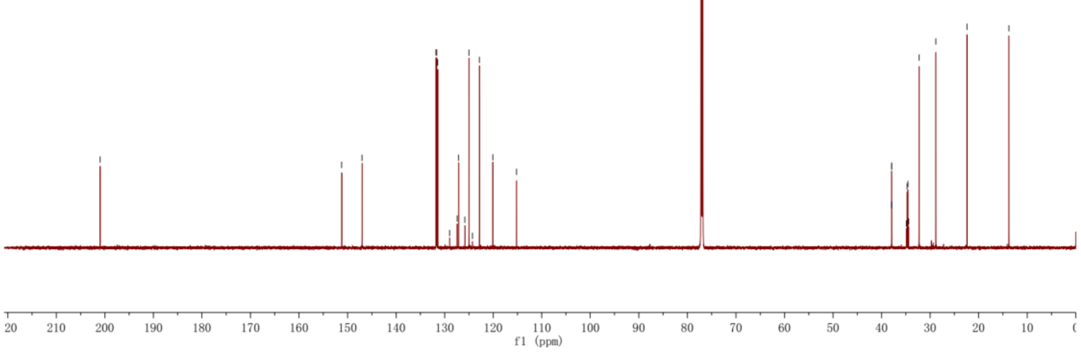

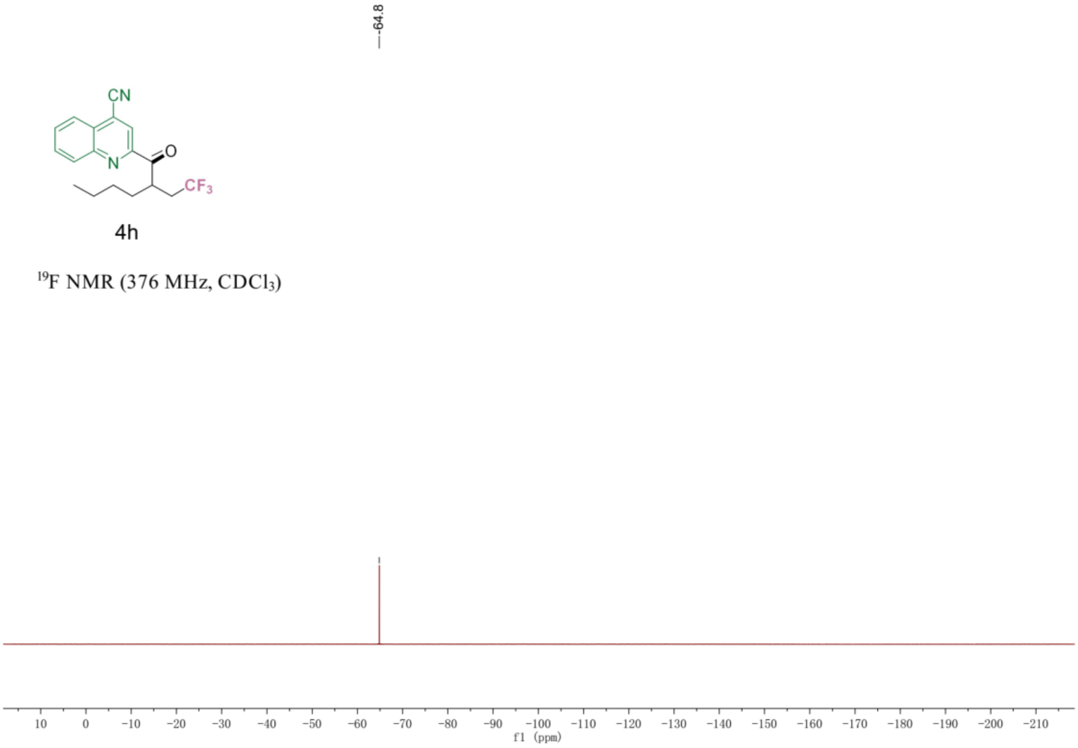

^{19}F NMR (376 MHz, $CDCl_3$)

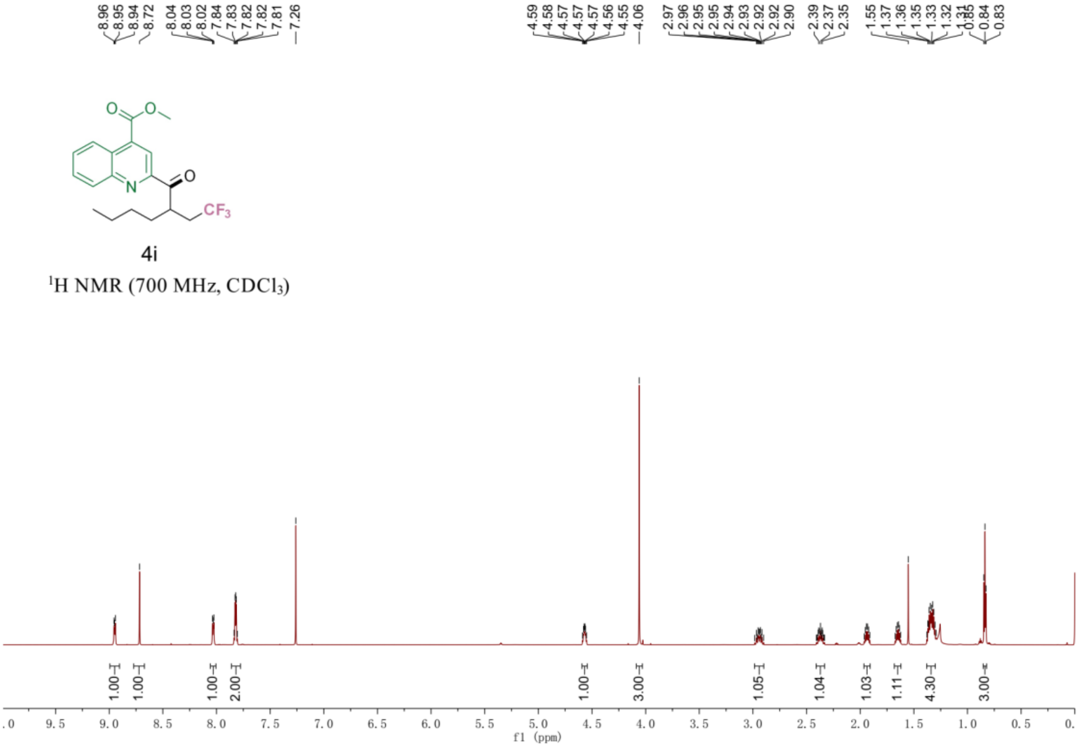

4i

^{1}H NMR (700 MHz, $CDCl_3$)

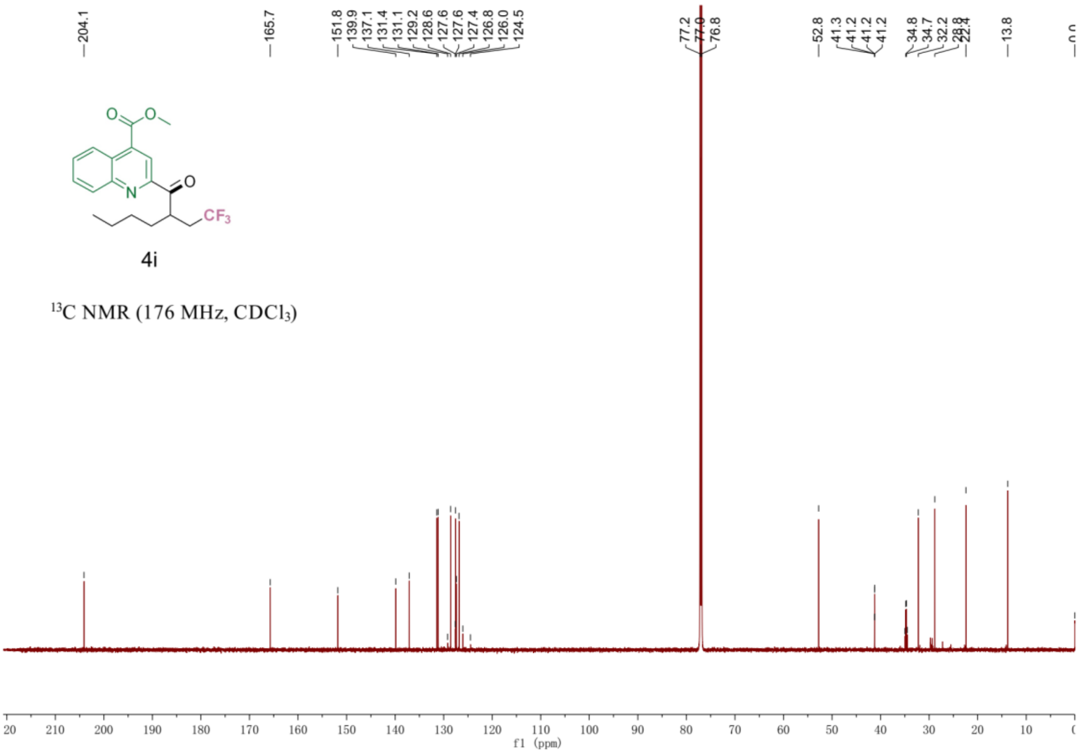

4i

^{13}C NMR (176 MHz, $CDCl_3$)

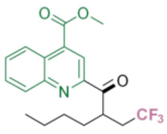

4i

^{19}F NMR (376 MHz, $CDCl_3$)

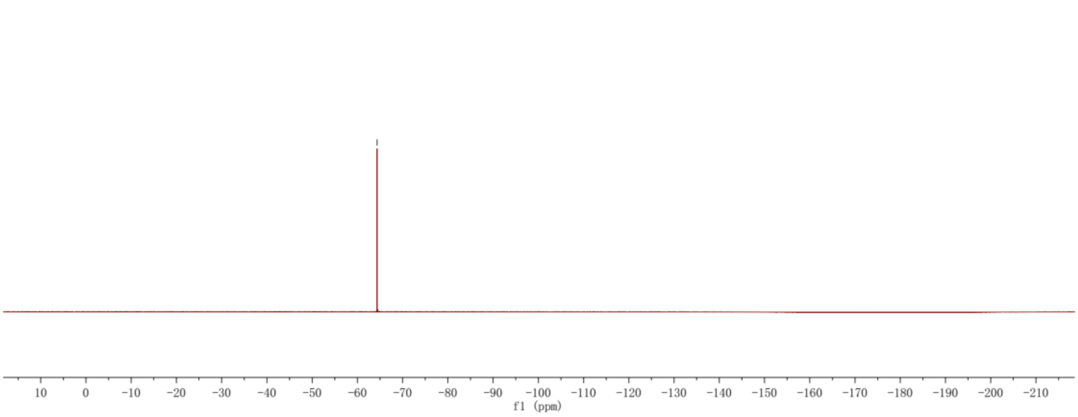

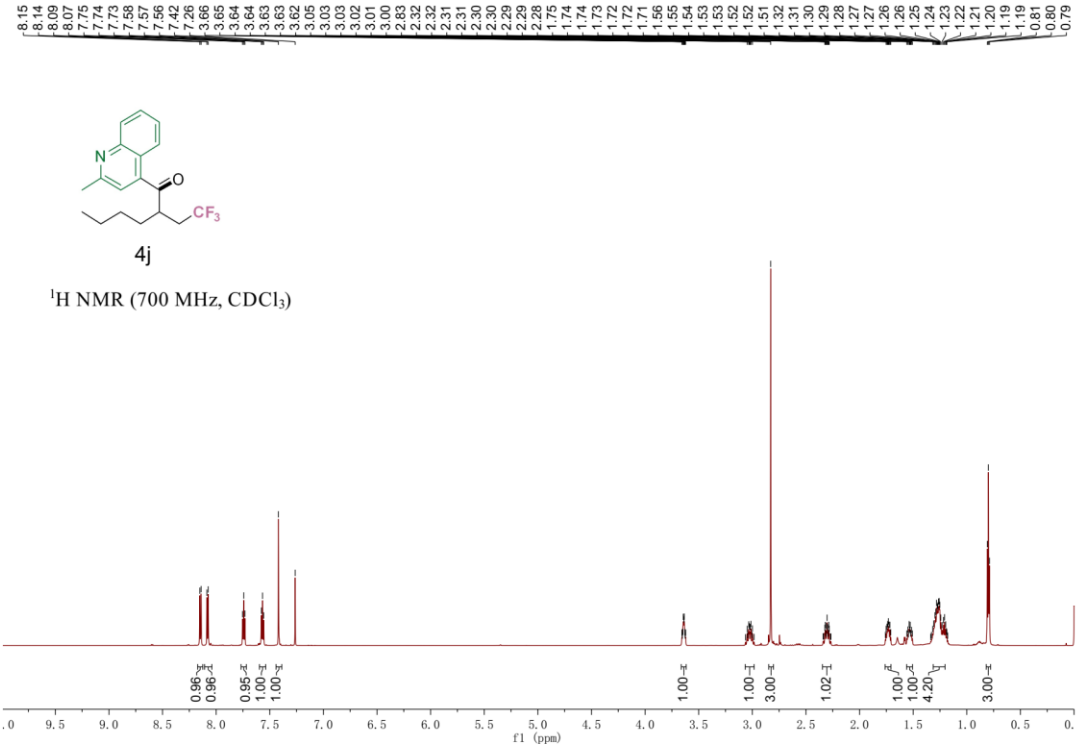

4j

^{1}H NMR (700 MHz, $CDCl_3$)

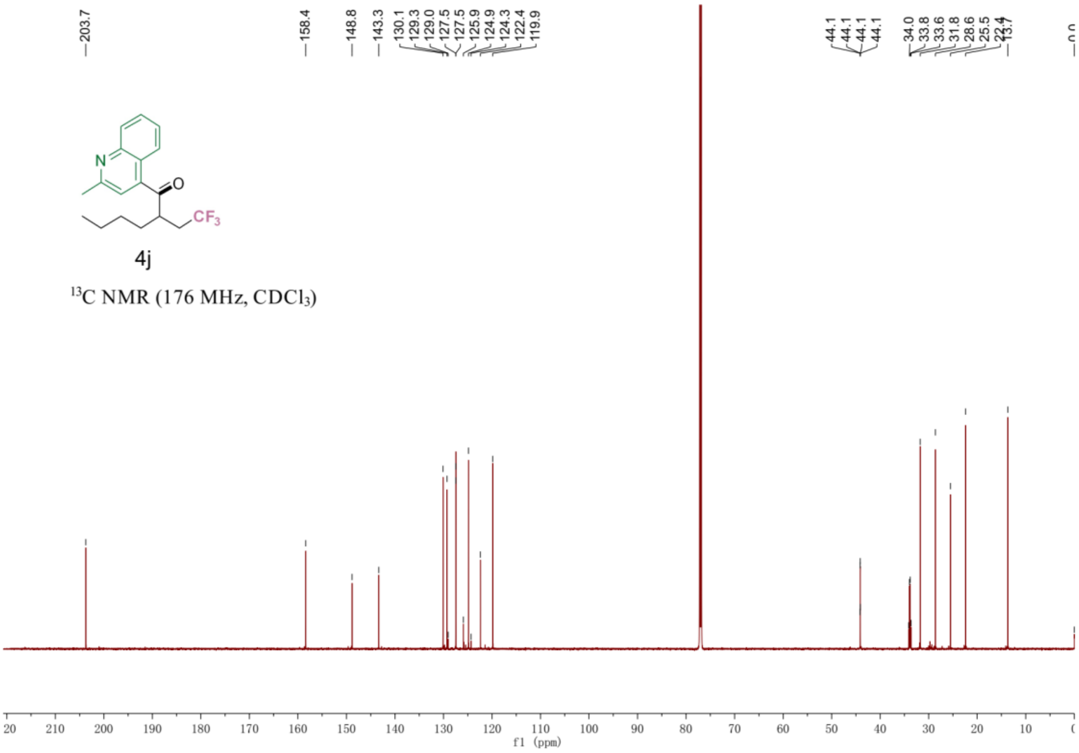

4j

^{13}C NMR (176 MHz, $CDCl_3$)

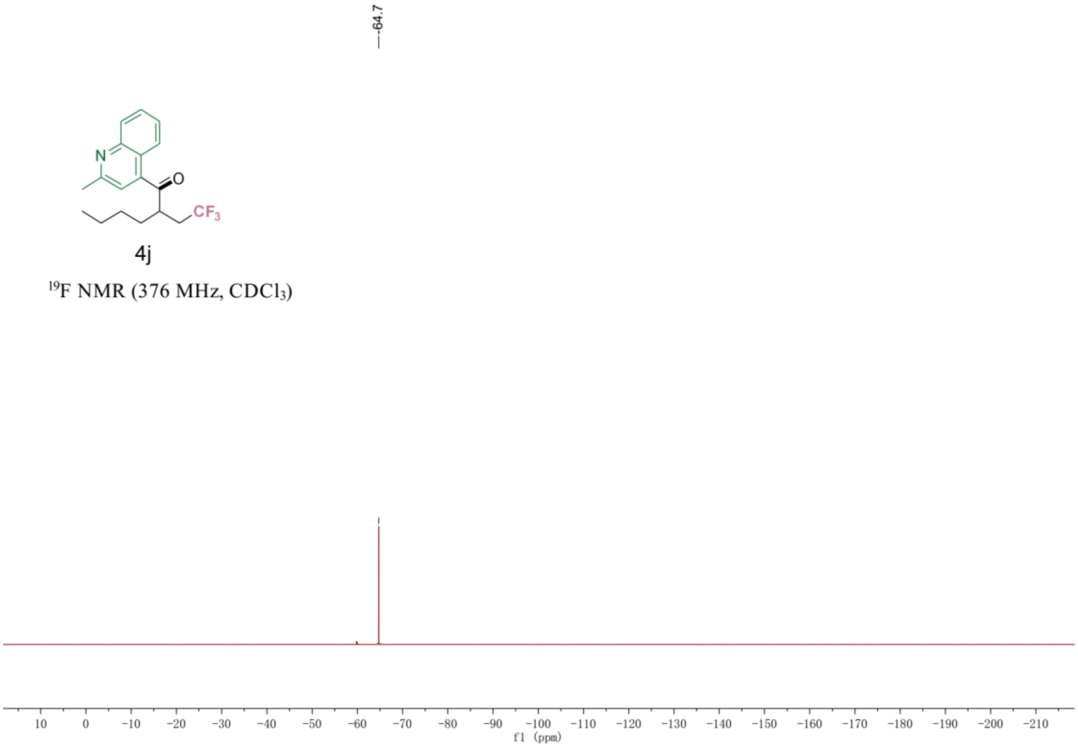

^{19}F NMR (376 MHz, $CDCl_3$)

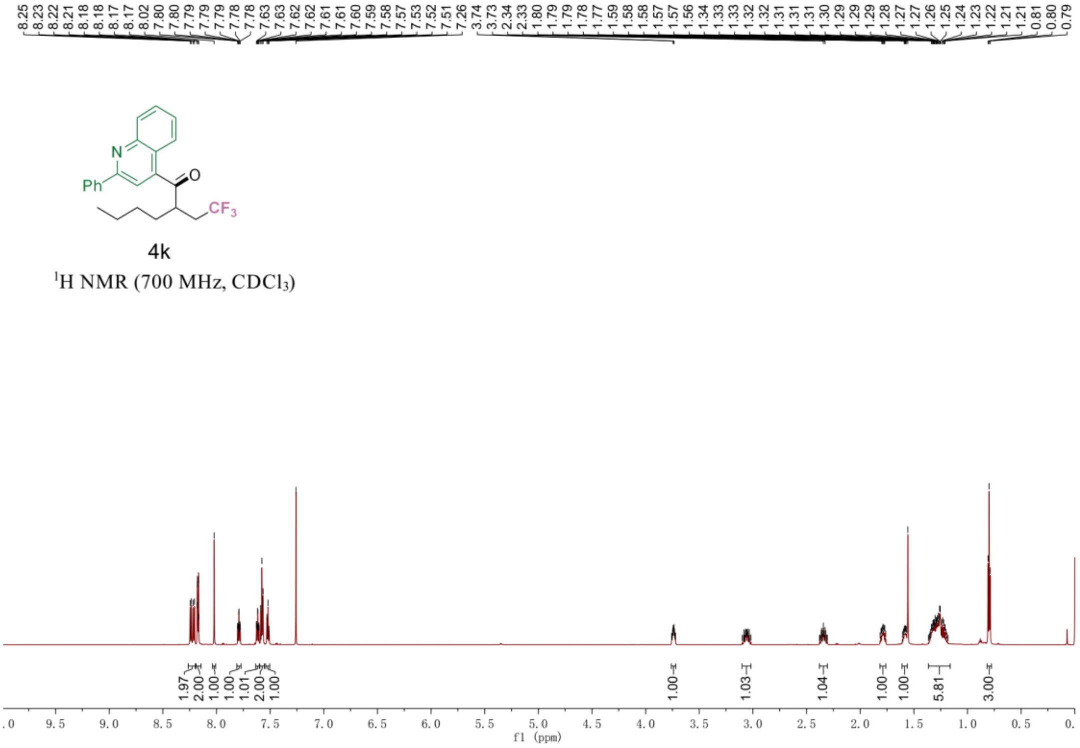

4k

^{1}H NMR (700 MHz, $CDCl_3$)

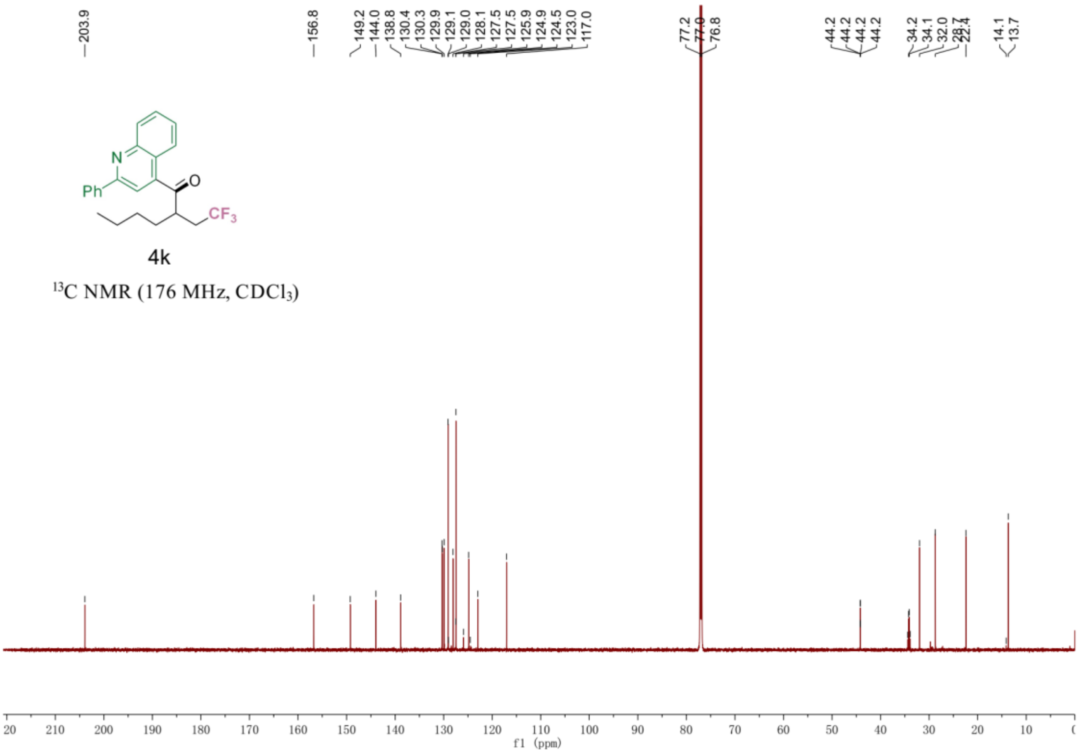

4k

^{13}C NMR (176 MHz, $CDCl_3$)

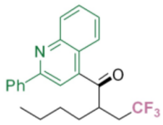

4k

^{19}F NMR (376 MHz, $CDCl_3$)

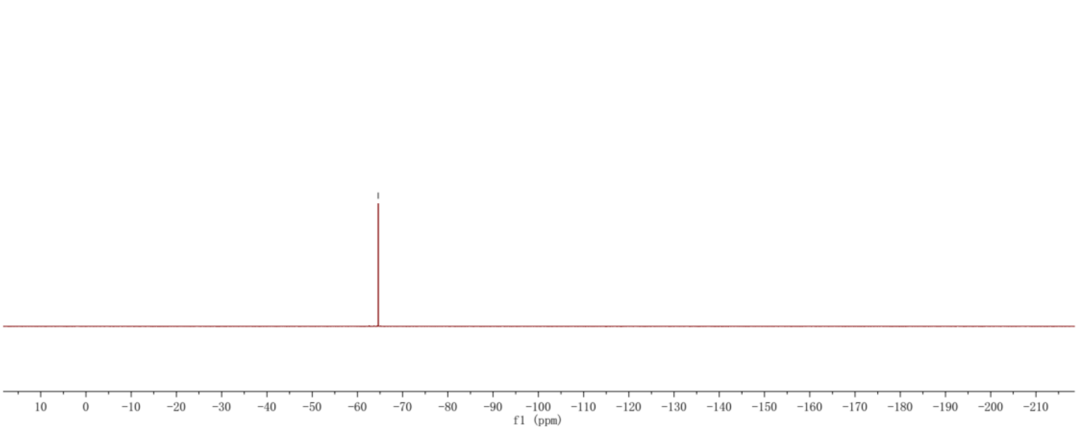

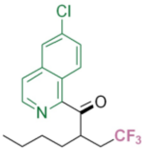

4l

^{1}H NMR (700 MHz, $CDCl_3$)

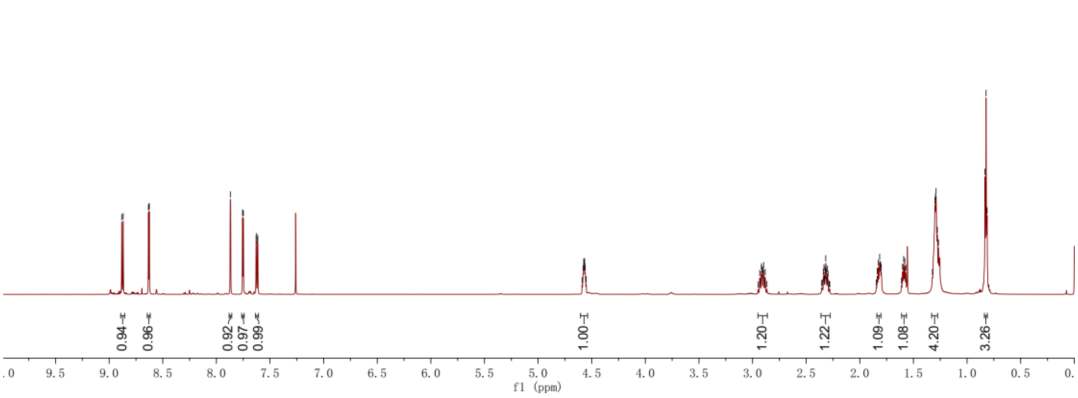

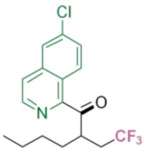

4l

^{13}C NMR (176 MHz, $CDCl_3$)

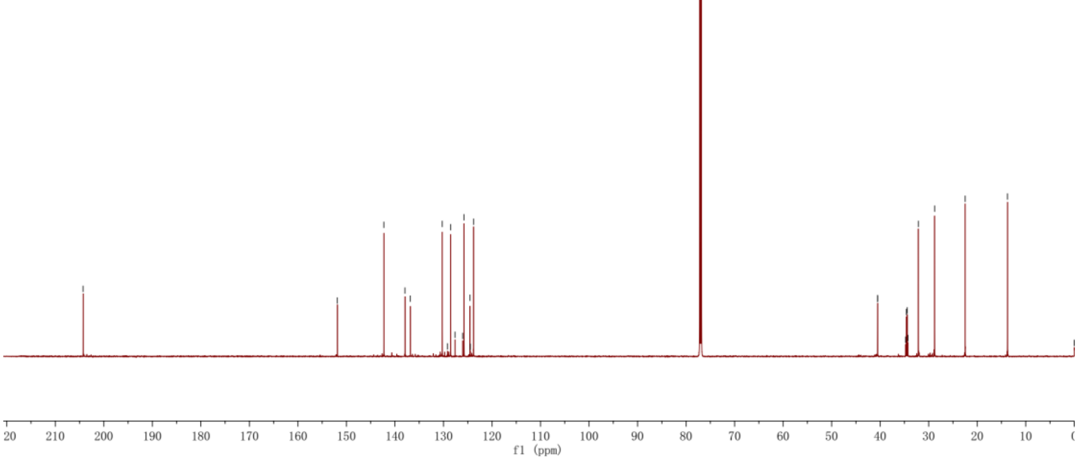

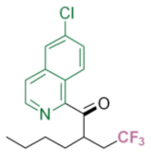

4l

^{19}F NMR (376 MHz, $CDCl_3$)

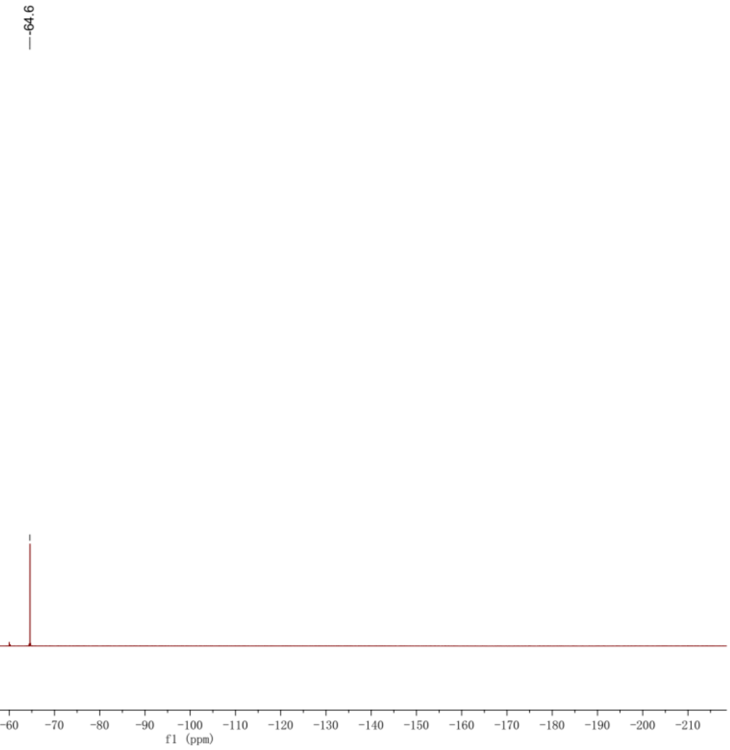

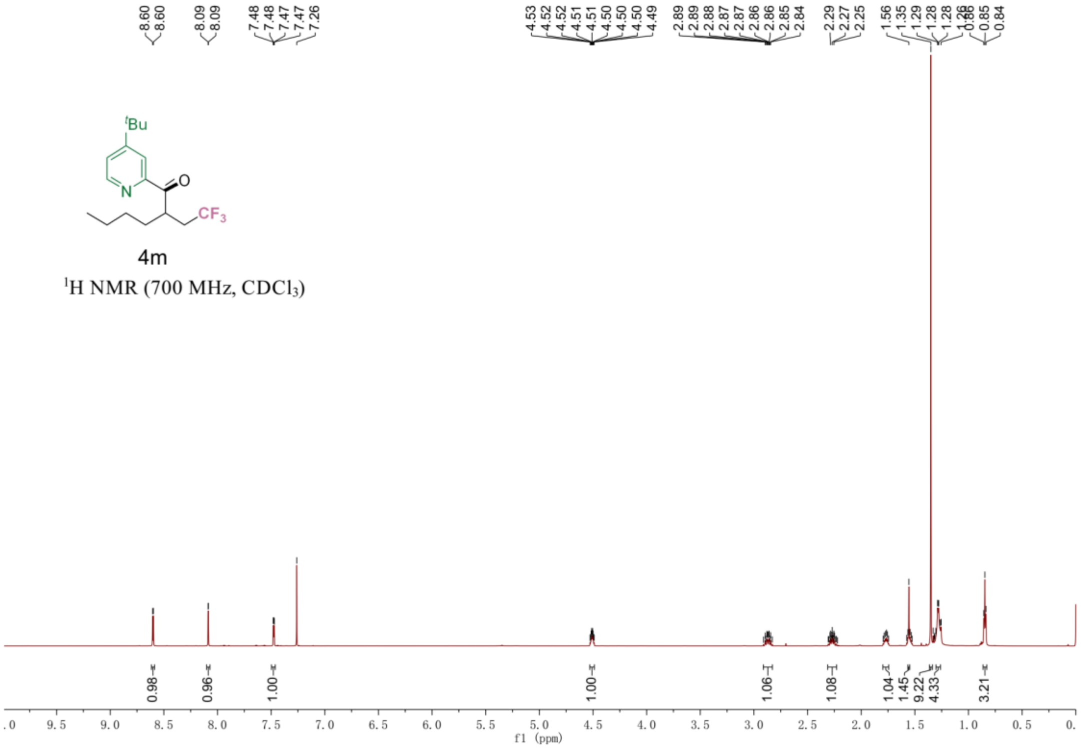

4m

^{1}H NMR (700 MHz, CDCl3)

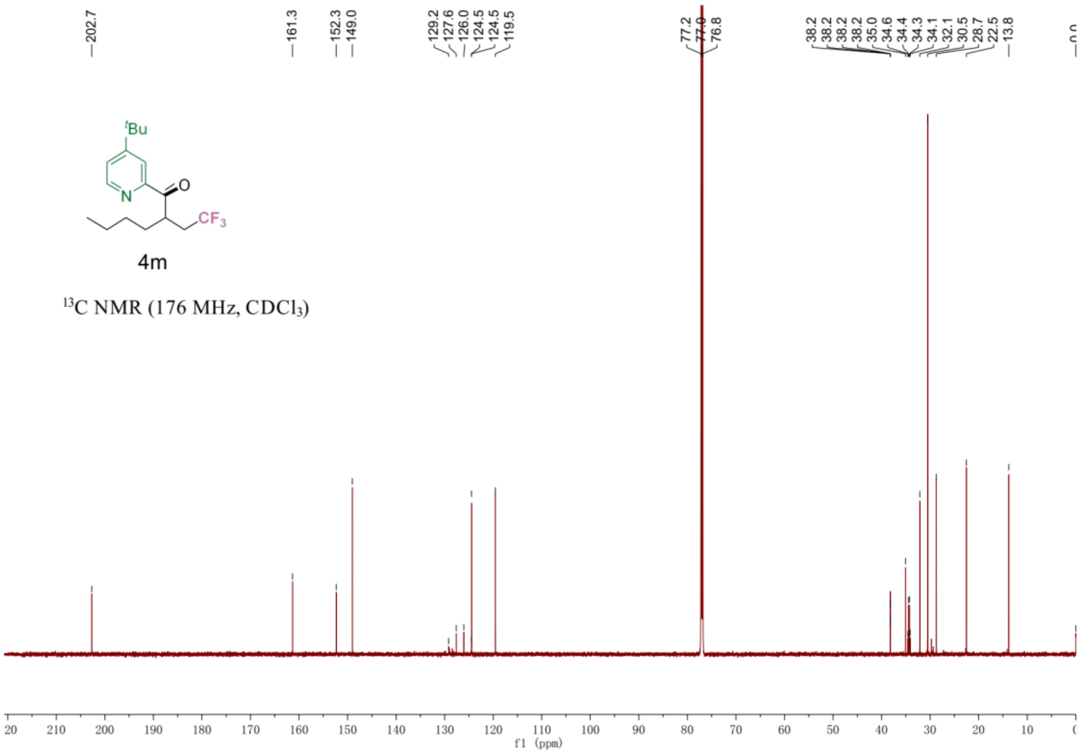

4m

^{13}C NMR (176 MHz, CDCl3)

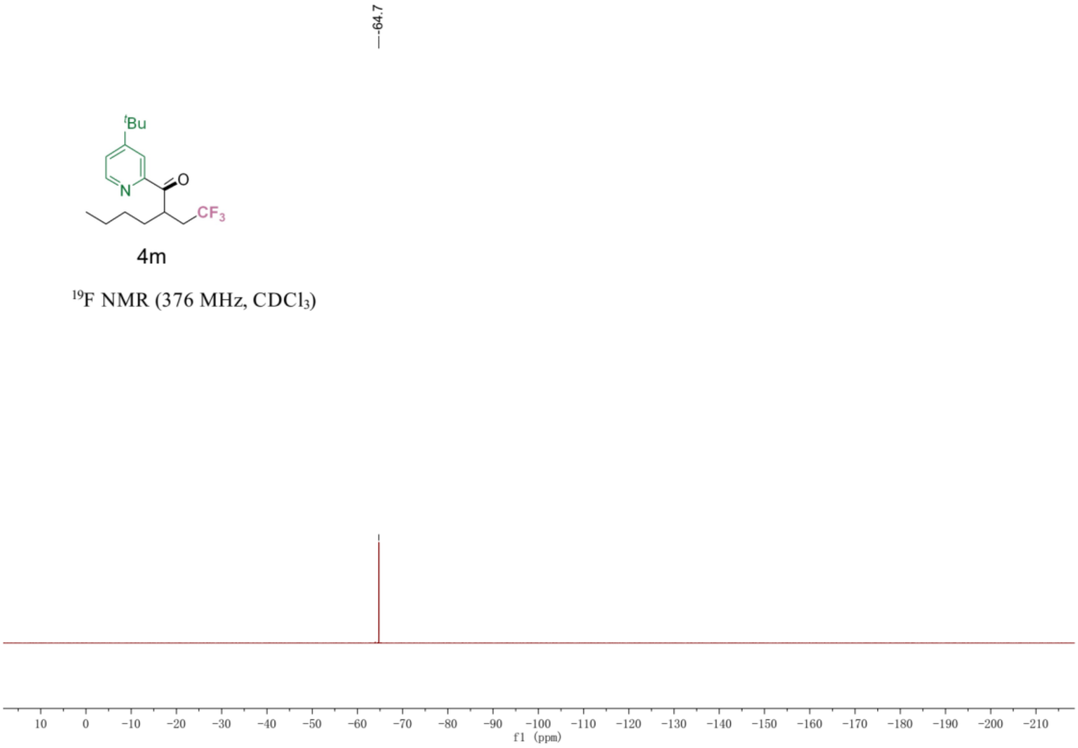

^{19}F NMR (376 MHz, $CDCl_3$)

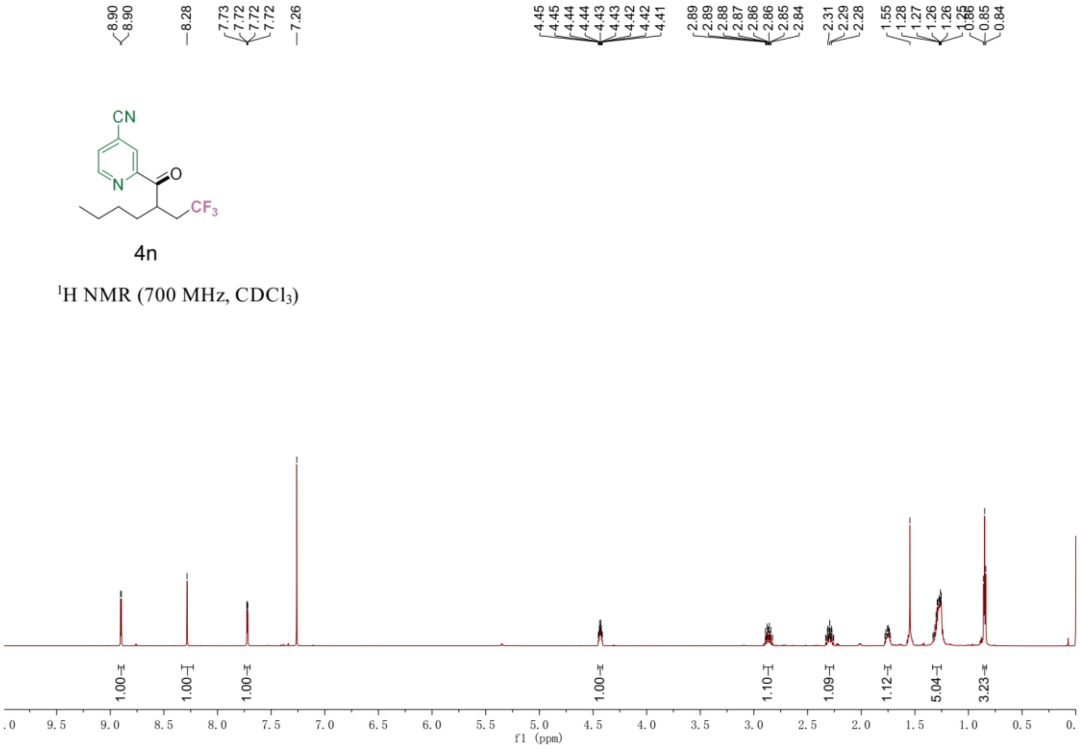

4n

^1H NMR (700 MHz, $CDCl_3$)

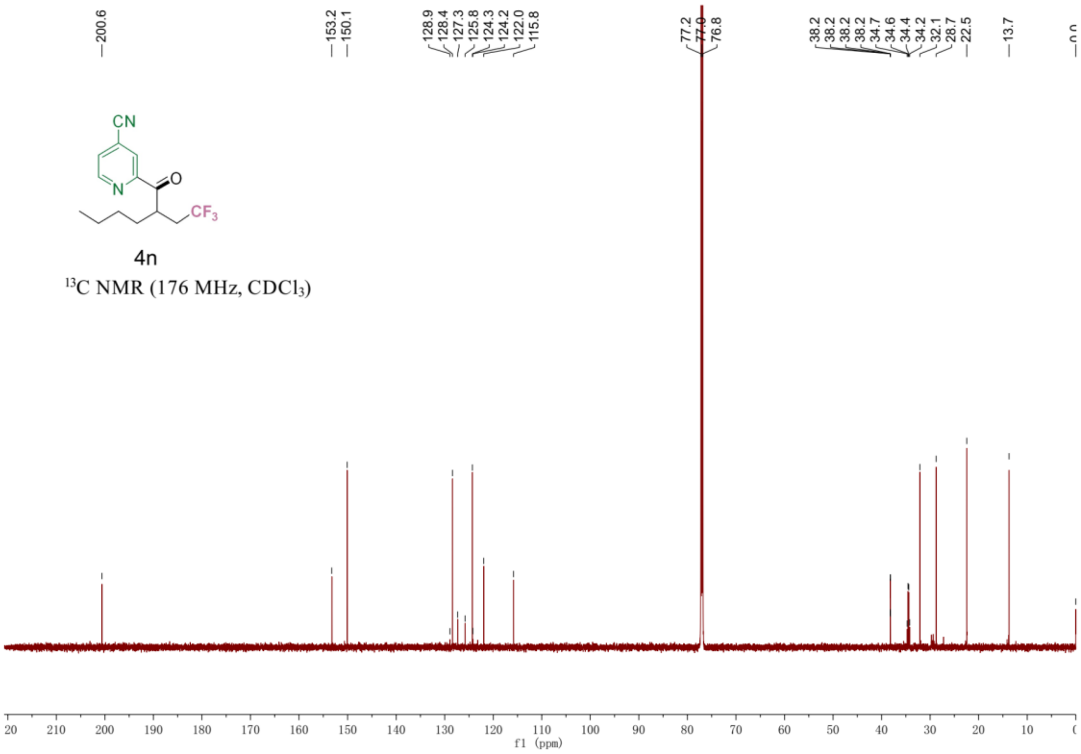

4n

^{13}C NMR (176 MHz, $CDCl_3$)

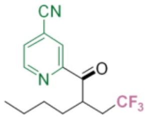

4n

^{19}F NMR (376 MHz, $CDCl_3$)

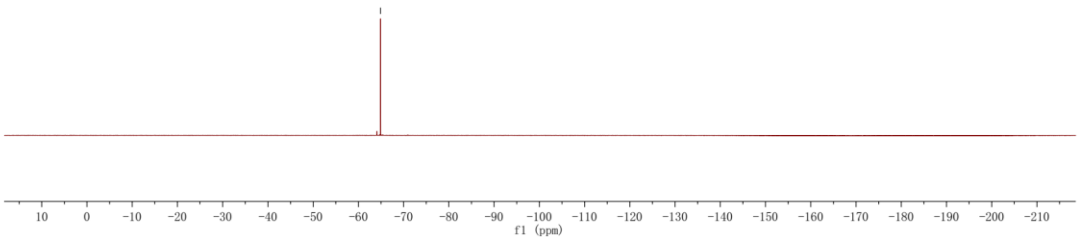

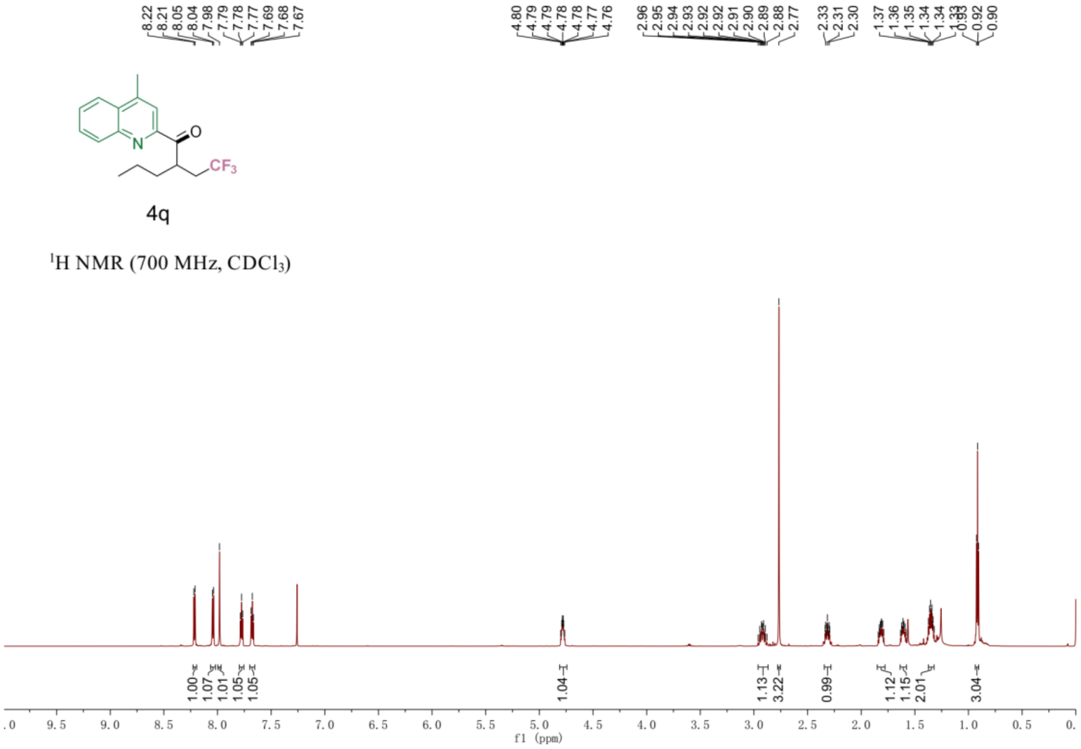

4q

^{1}H NMR (700 MHz, $CDCl_3$)

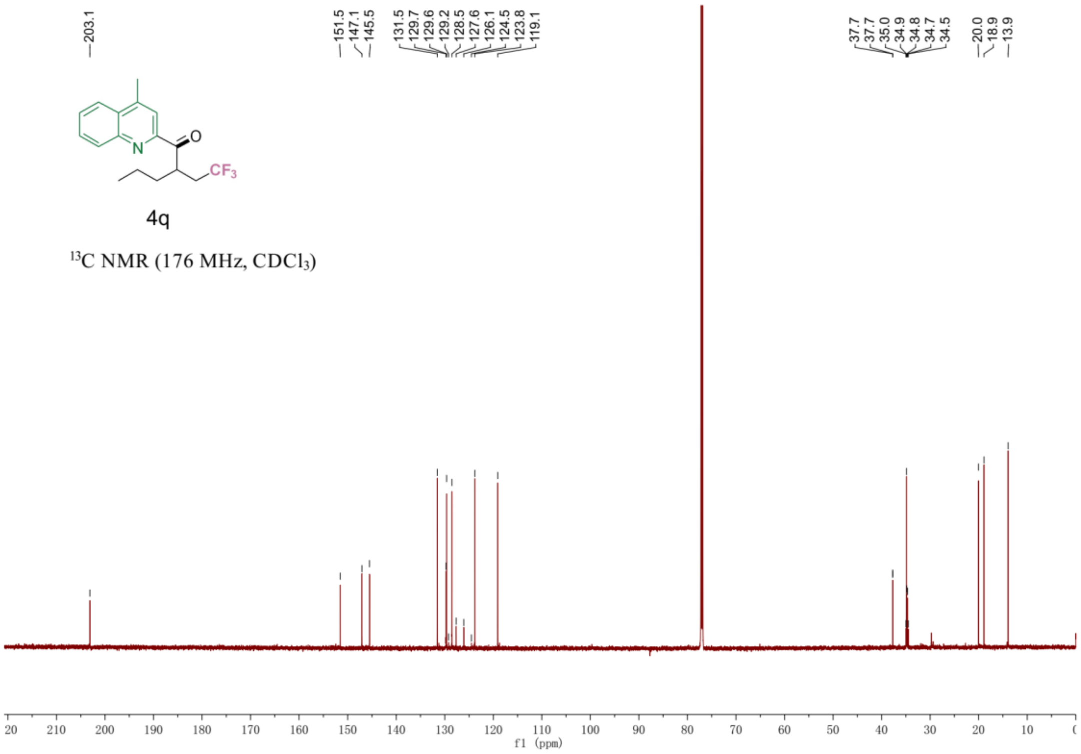

4q

^{13}C NMR (176 MHz, $CDCl_3$)

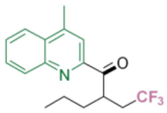

4q

^{19}F NMR (376 MHz, $CDCl_3$)

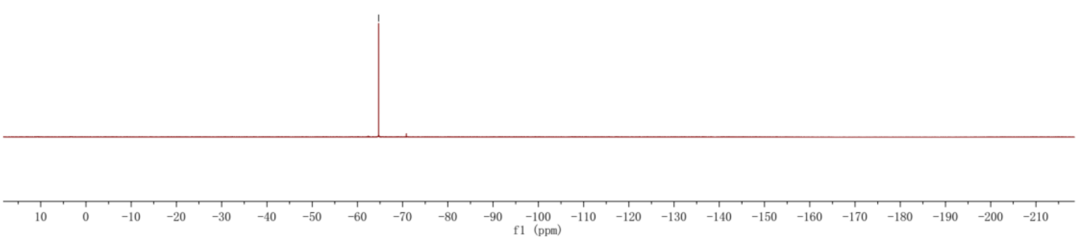

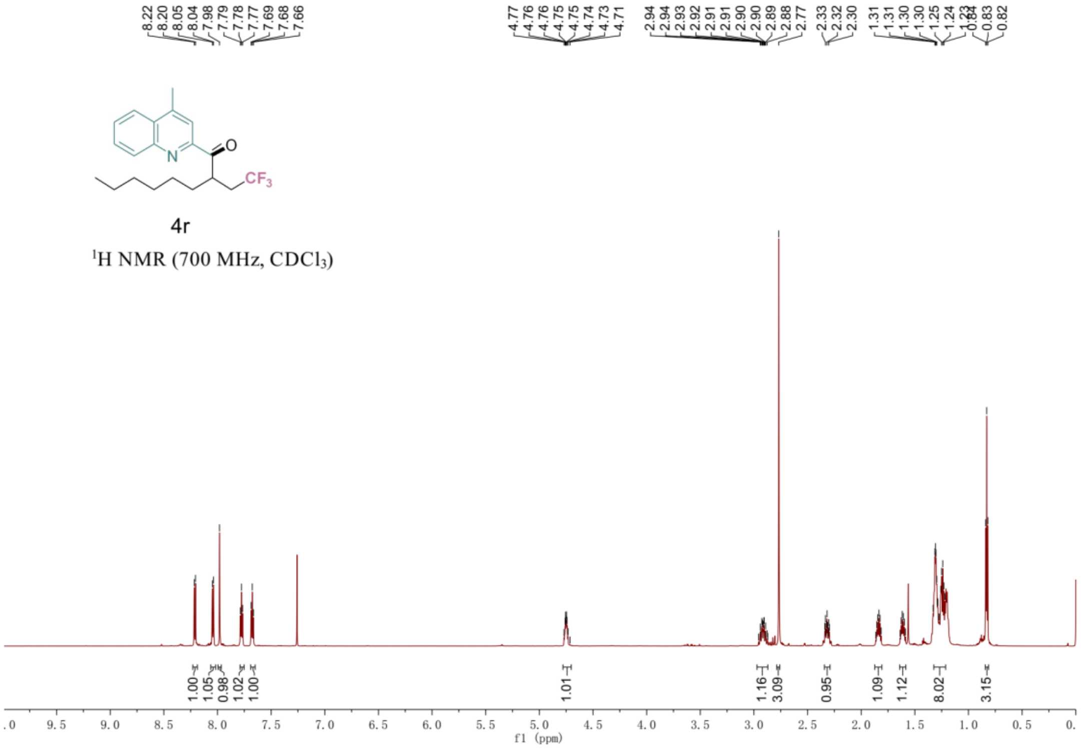

4r

^{1}H NMR (700 MHz, $CDCl_3$)

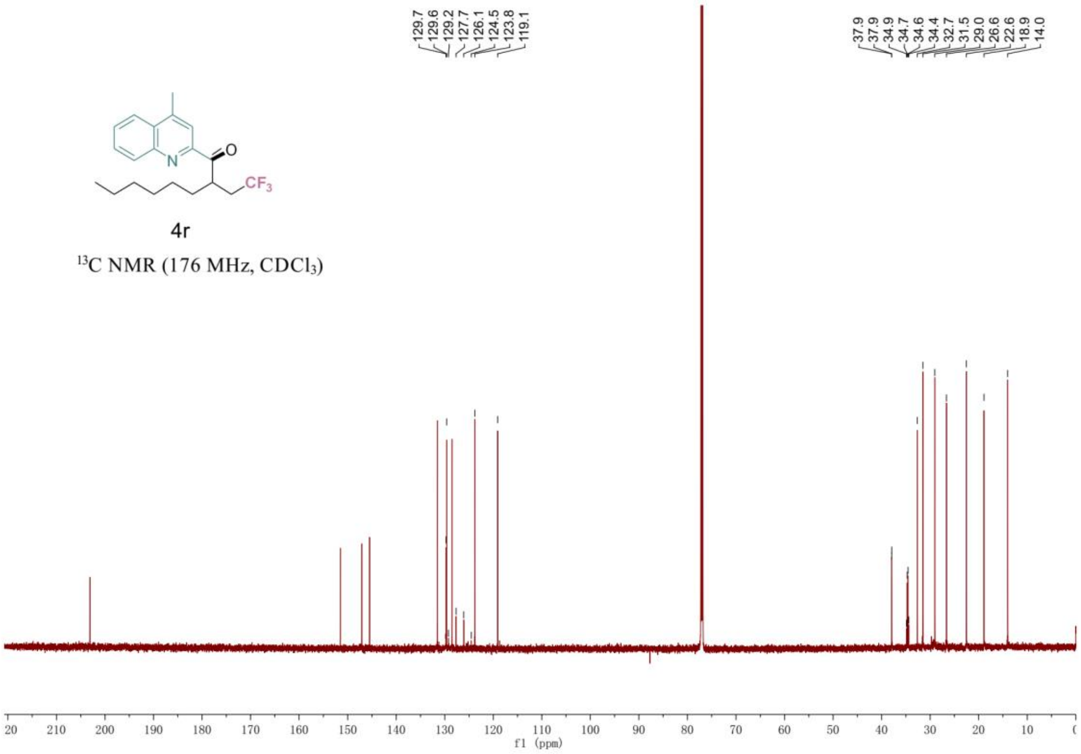

4r

^{13}C NMR (176 MHz, $CDCl_3$)

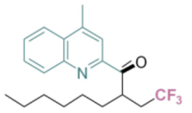

4r

^{19}F NMR (376 MHz, $CDCl_3$)

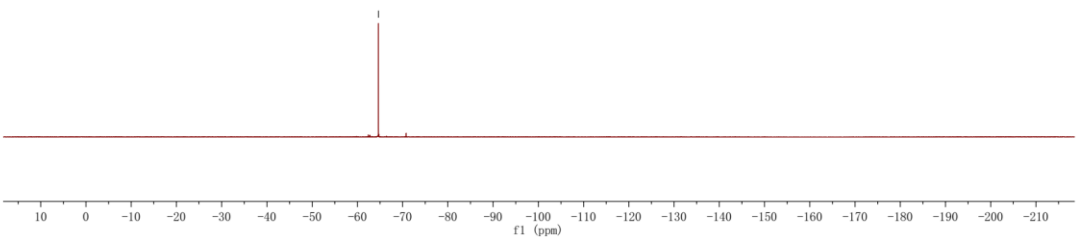

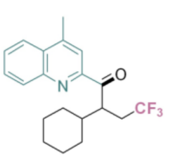

4s

^{1}H NMR (700 MHz, CDCl$_3$)

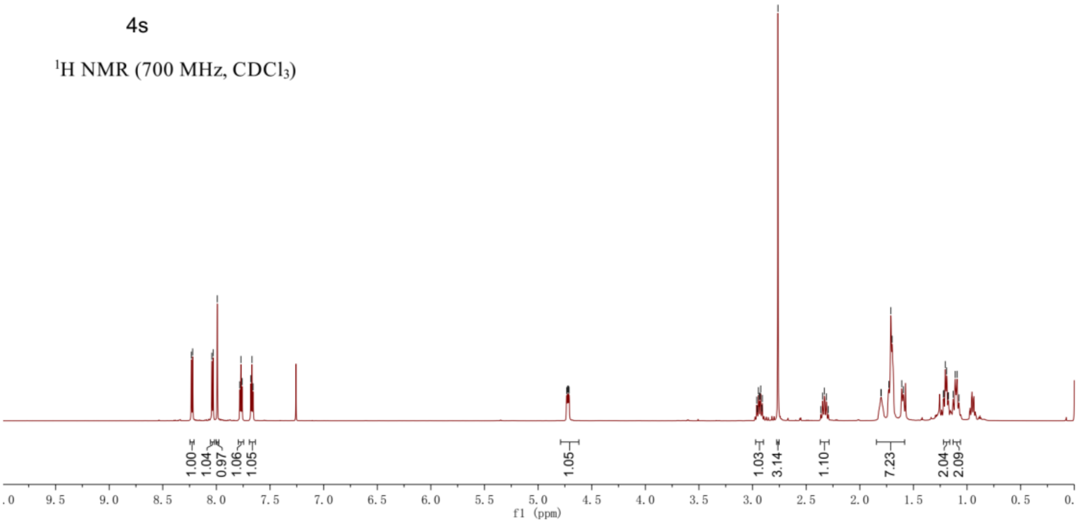

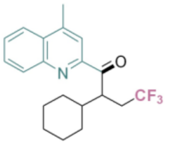

4s

^{13}C NMR (176 MHz, CDCl$_3$)

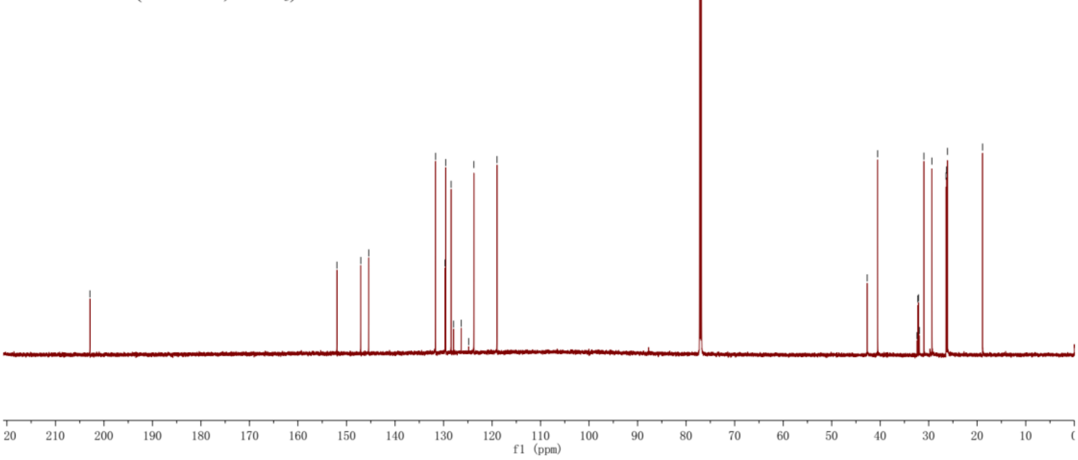

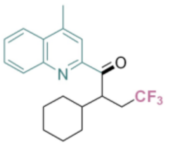

4s

^{19}F NMR (376 MHz, $CDCl_3$)

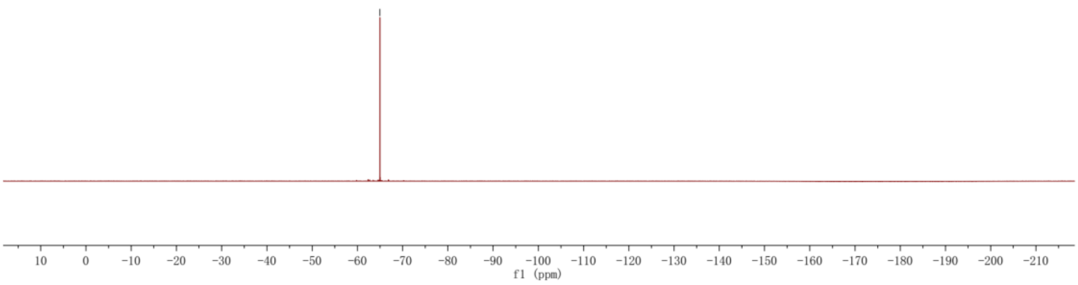

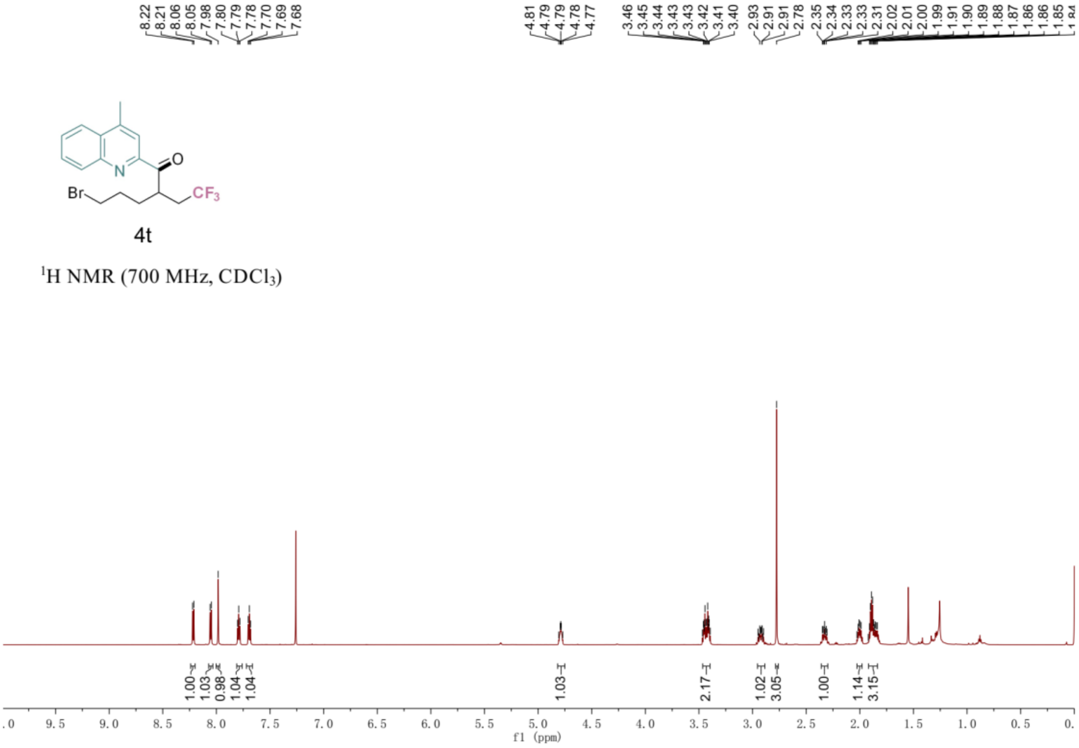

4t

^{1}H NMR (700 MHz, $CDCl_3$)

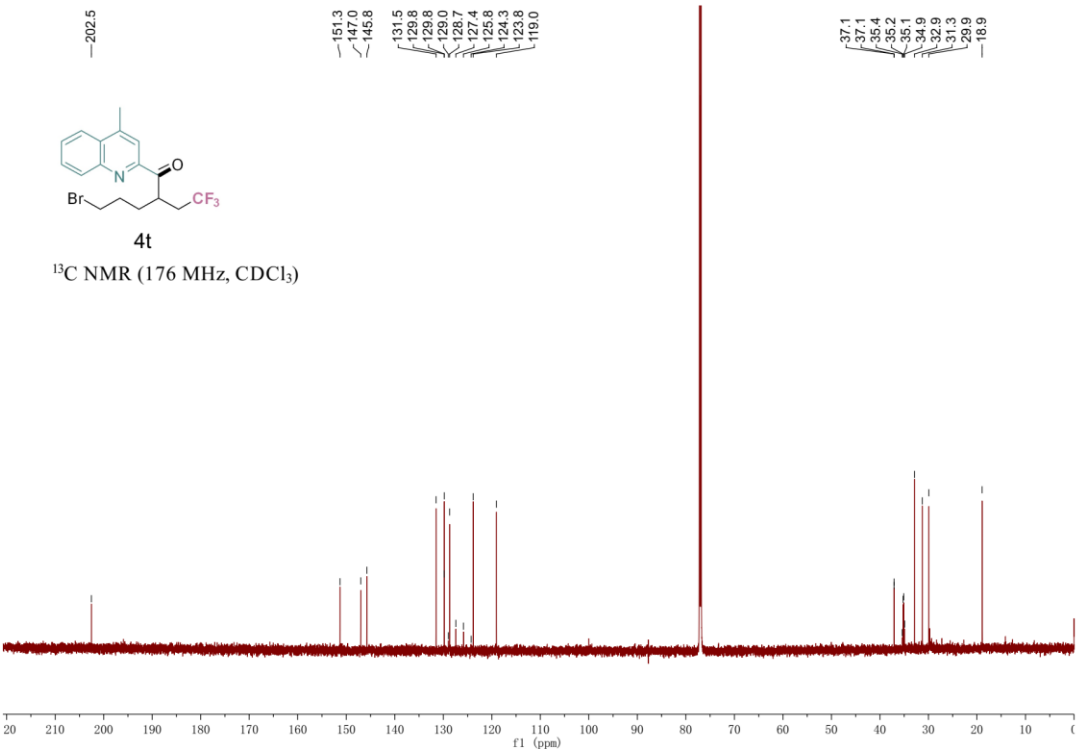

4t

^{13}C NMR (176 MHz, $CDCl_3$)

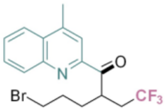

4t

^{19}F NMR (376 MHz, $CDCl_3$)

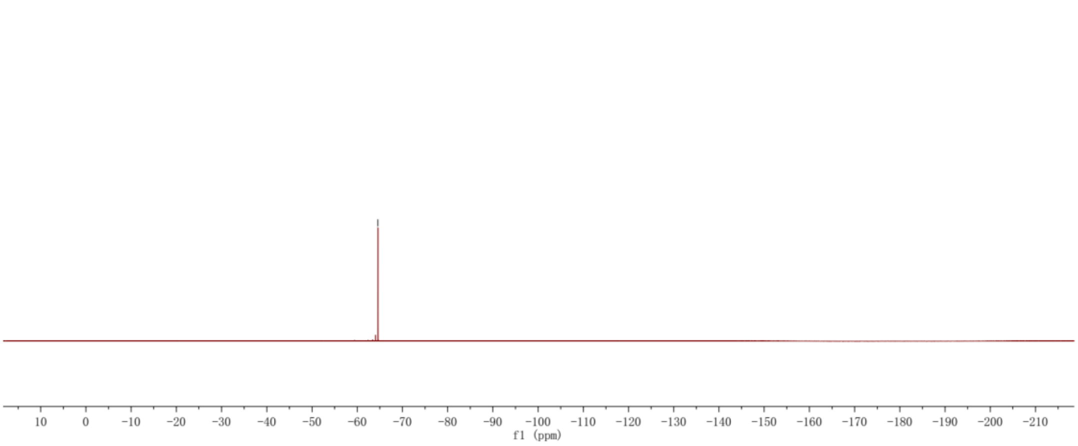

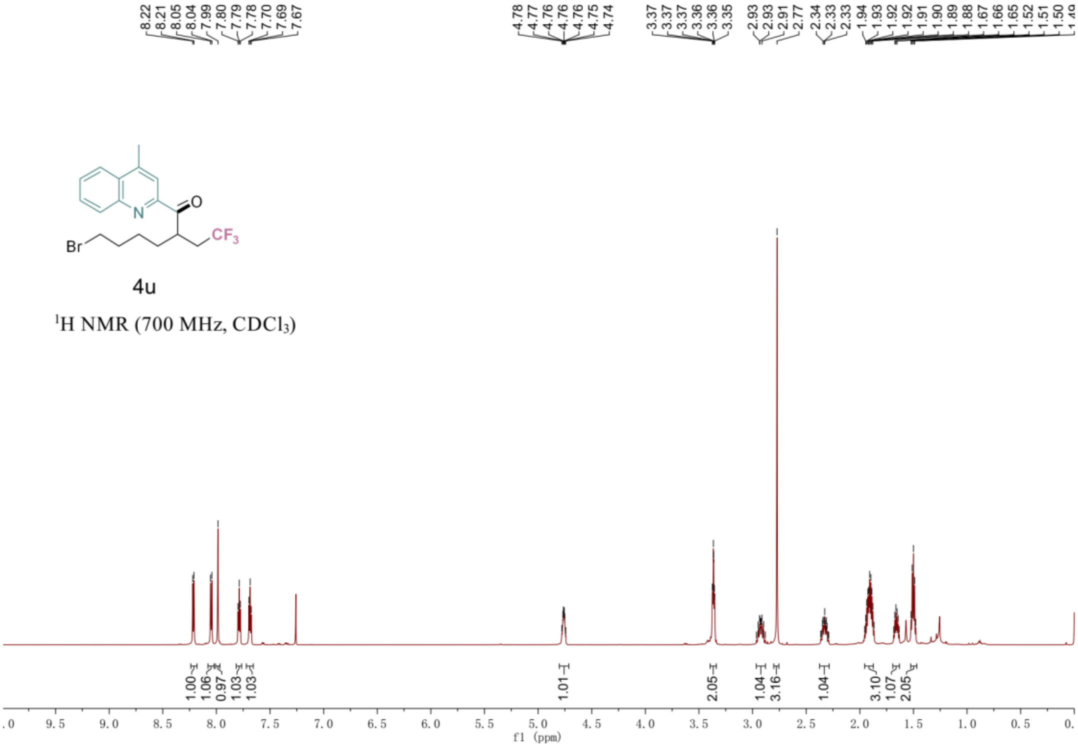

4u

^{1}H NMR (700 MHz, $CDCl_3$)

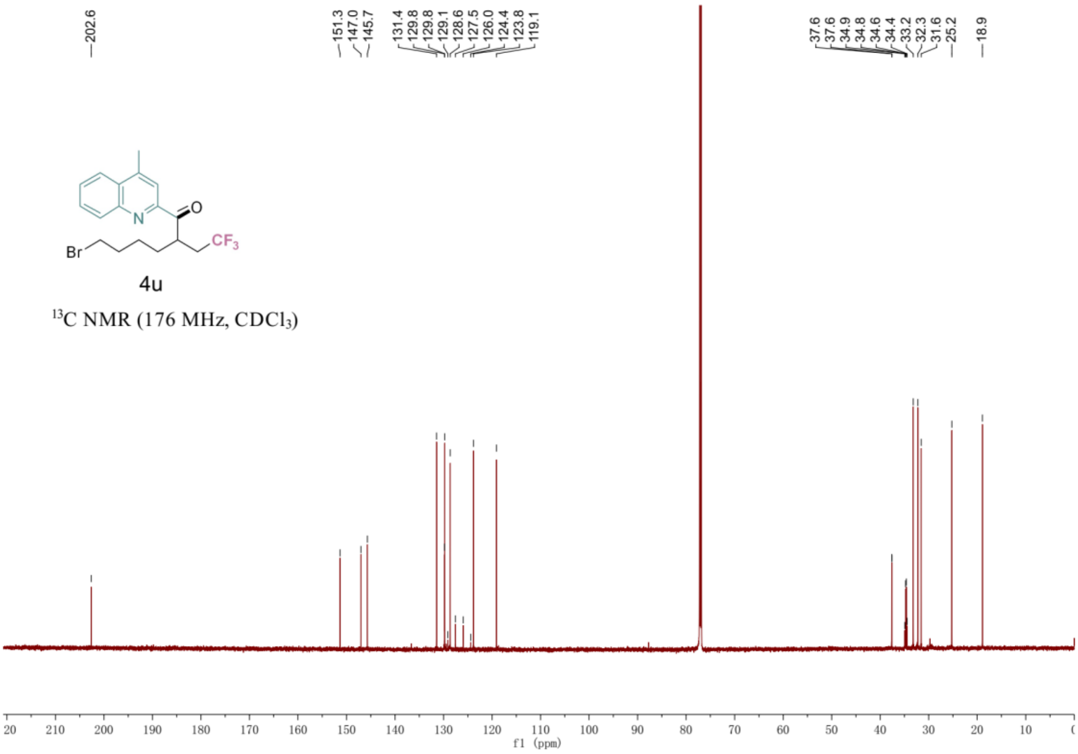

4u

^{13}C NMR (176 MHz, $CDCl_3$)

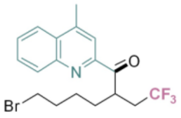

4u

^{19}F NMR (376 MHz, $CDCl_3$)

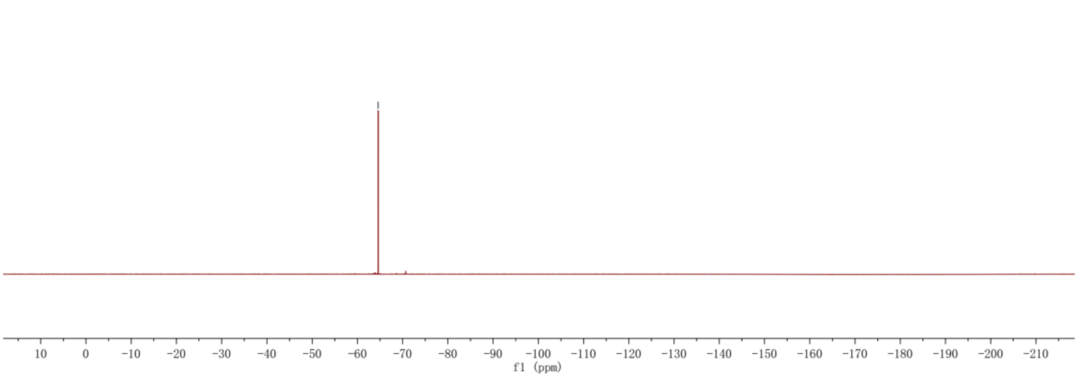

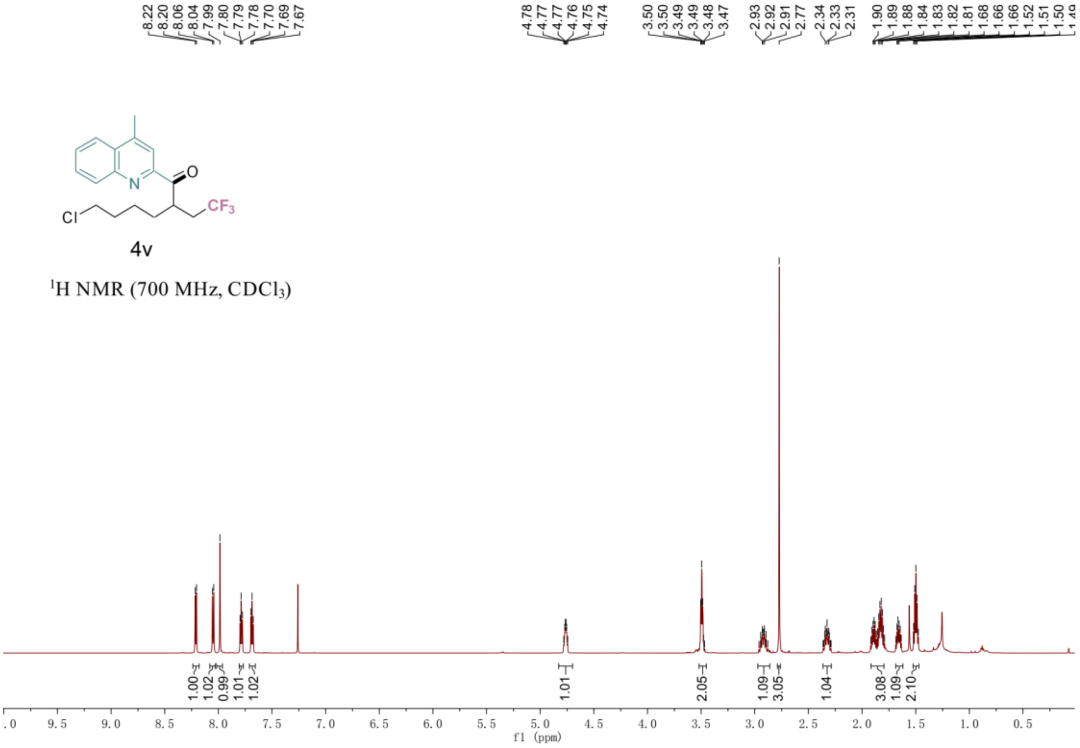

4v

^{1}H NMR (700 MHz, $CDCl_3$)

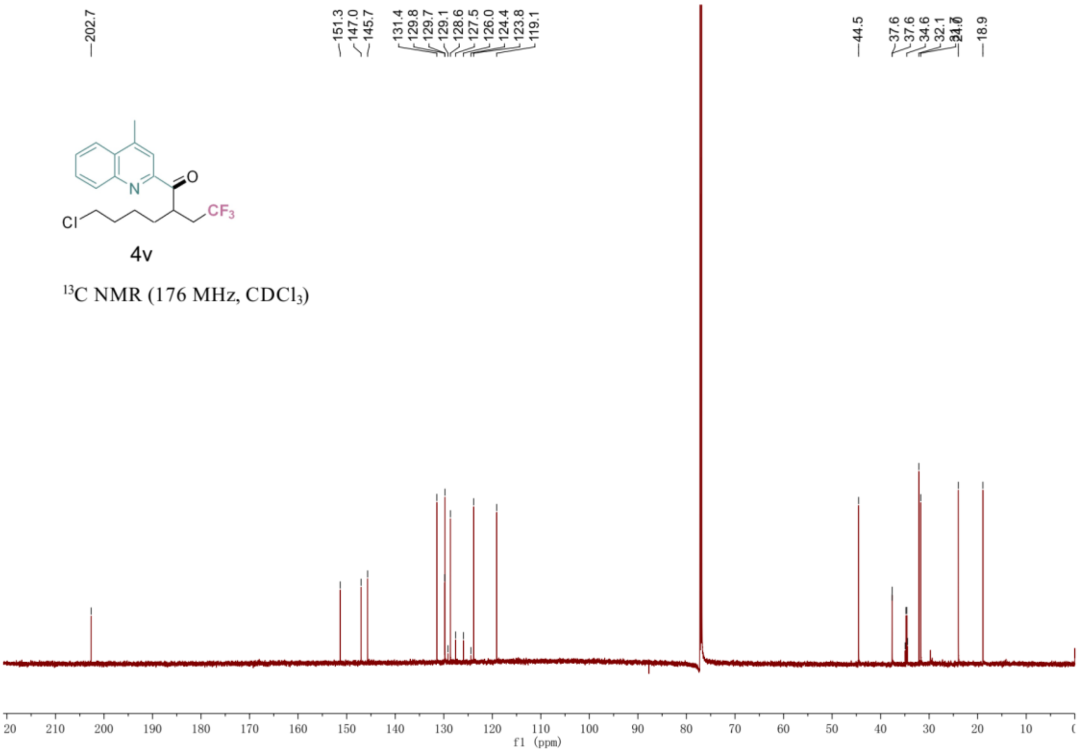

4v

^{13}C NMR (176 MHz, $CDCl_3$)

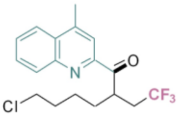

4v

^{19}F NMR (376 MHz, $CDCl_3$)

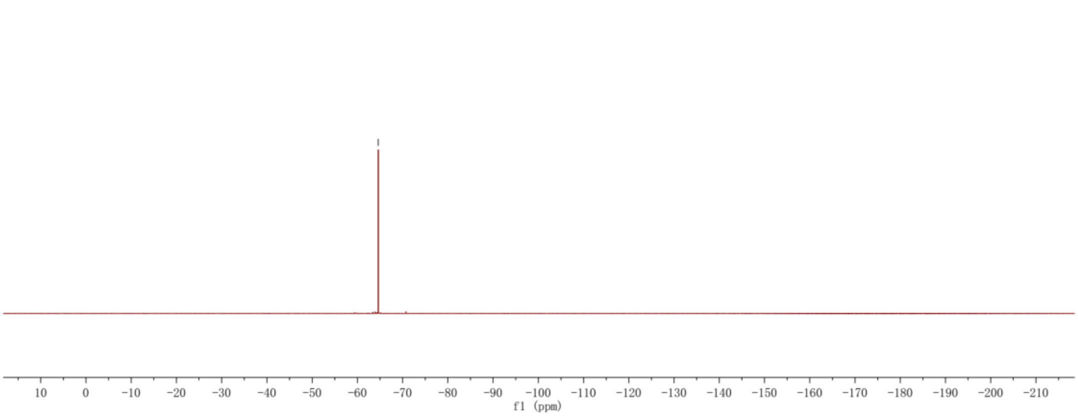

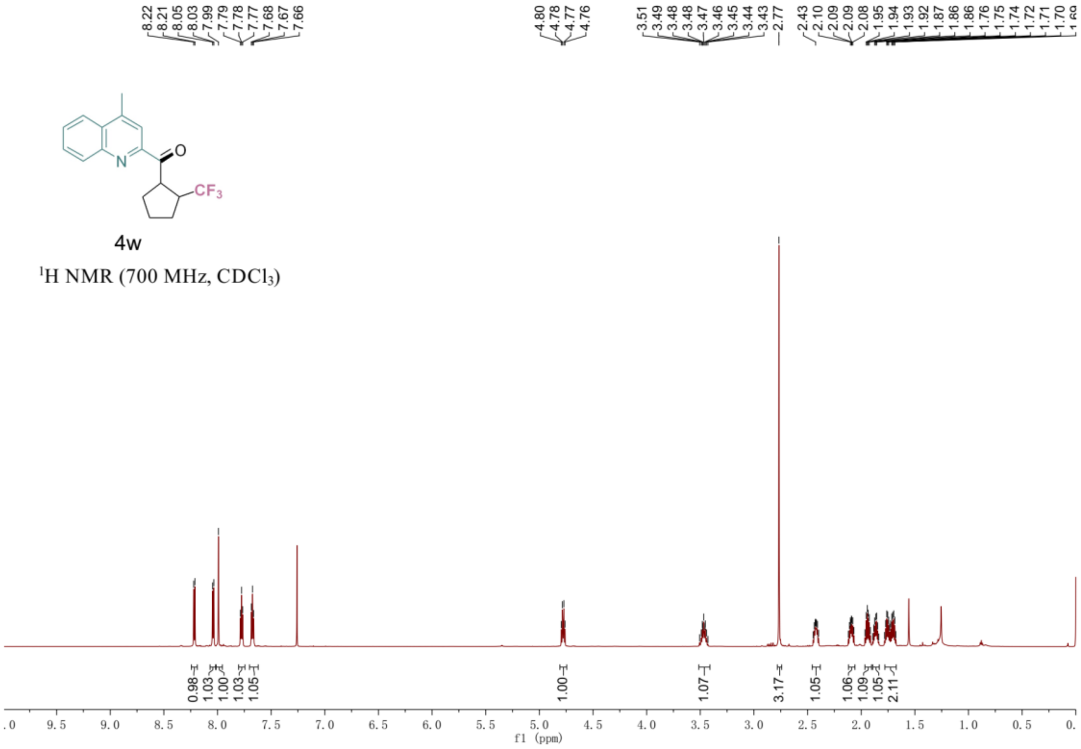
8.22
8.21
8.05
8.03
7.99
7.79
7.78
7.77
7.68
7.67
7.66
4.80
4.78
4.77
4.76
3.51
3.49
3.48
3.47
3.46
3.45
3.44
3.43
2.77
2.43
2.10
2.09
2.09
2.08
1.95
1.94
1.93
1.92
1.87
1.86
1.86
1.76
1.75
1.74
1.72
1.71
1.70
O
N
CF3
4w
1H NMR (700 MHz, CDCl3)
0.98
1.03
1.00
1.03
1.05
1.00
1.07
3.17
1.05
1.06
1.09
1.05
2.11
f1 (ppm)

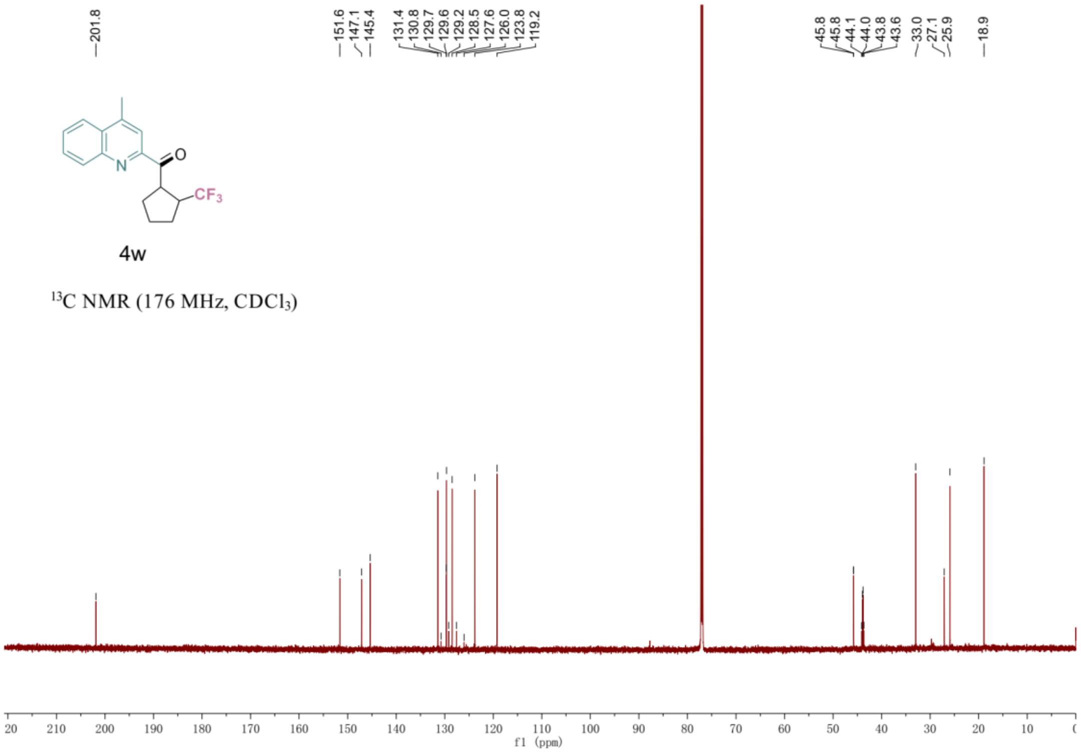
201.8
151.6
147.1
145.4
131.4
130.8
129.7
129.6
129.2
128.5
127.6
126.0
123.8
119.2
45.8
45.8
44.1
44.0
43.8
43.6
33.0
27.1
25.9
18.9
O
N
CF3
4w
13C NMR (176 MHz, CDCl3)
f1 (ppm)

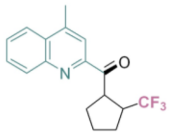

4w

^{19}F NMR (376 MHz, $CDCl_3$)

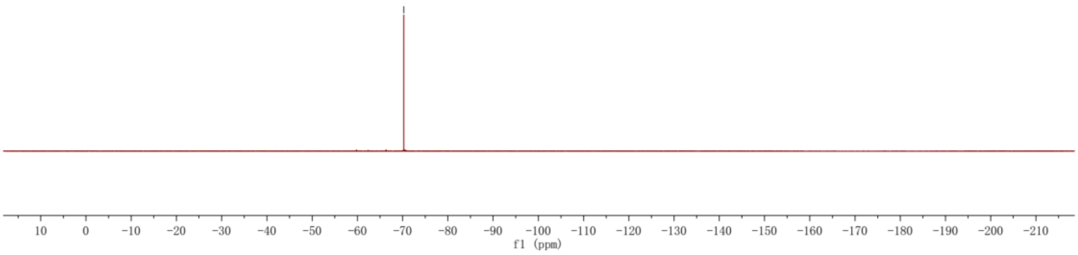

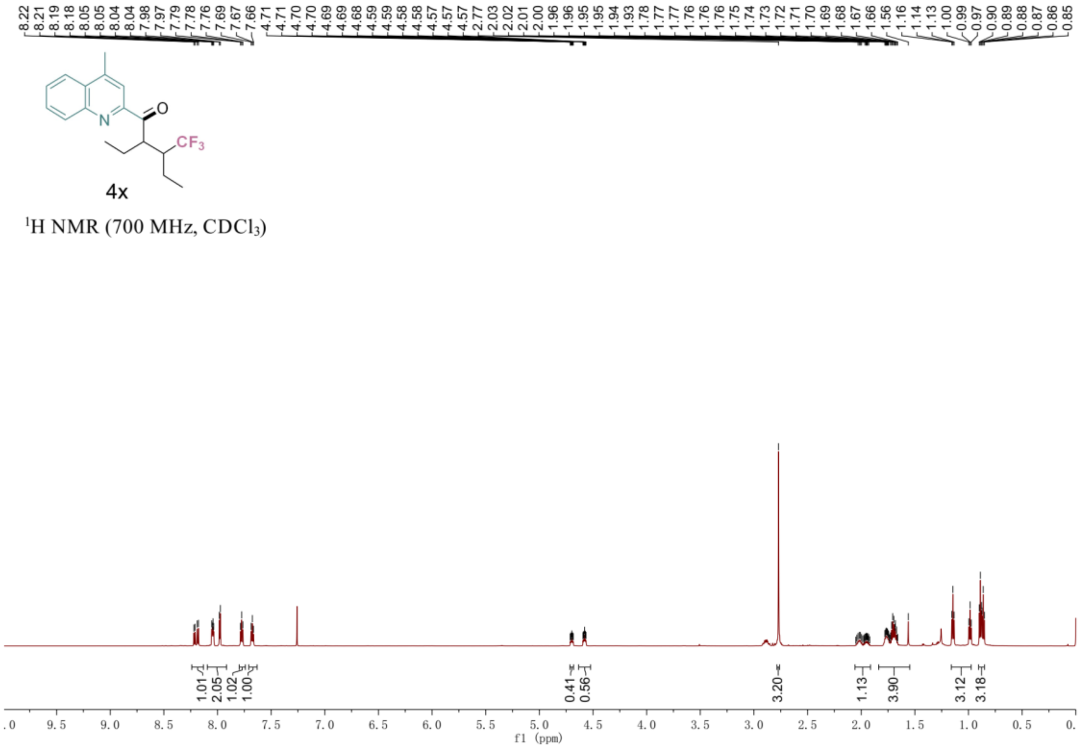

4x

^{1}H NMR (700 MHz, $CDCl_3$)

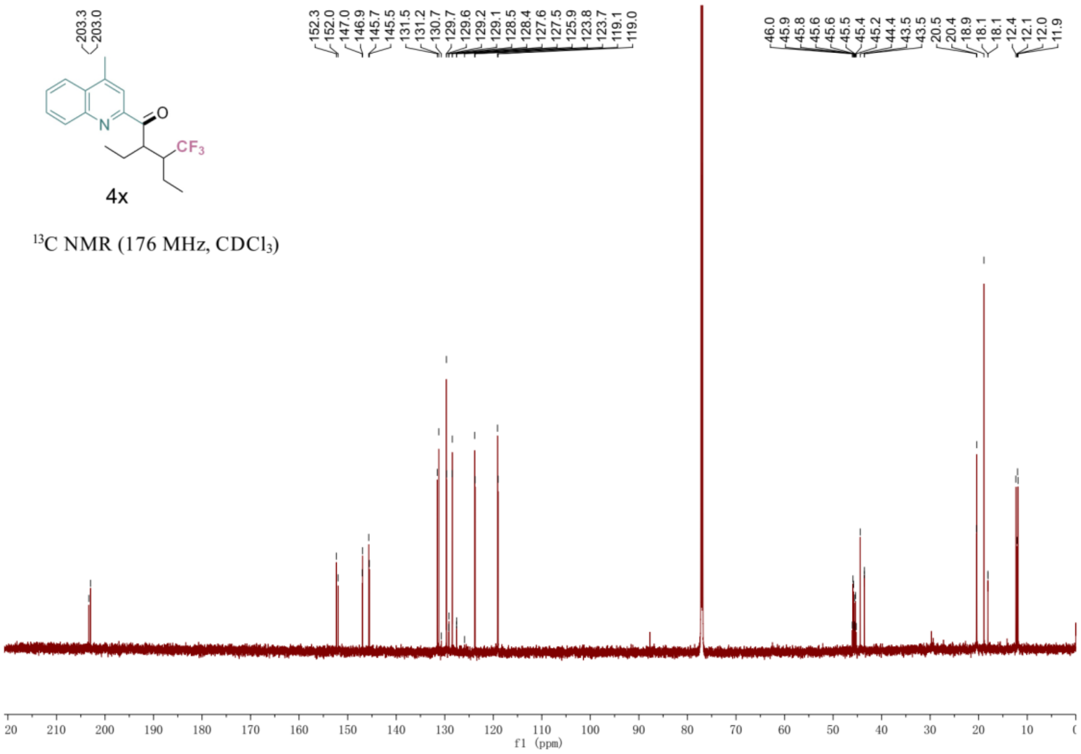

4x

^{13}C NMR (176 MHz, $CDCl_3$)

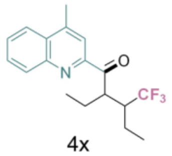

4x

^{19}F NMR (376 MHz, $CDCl_3$)

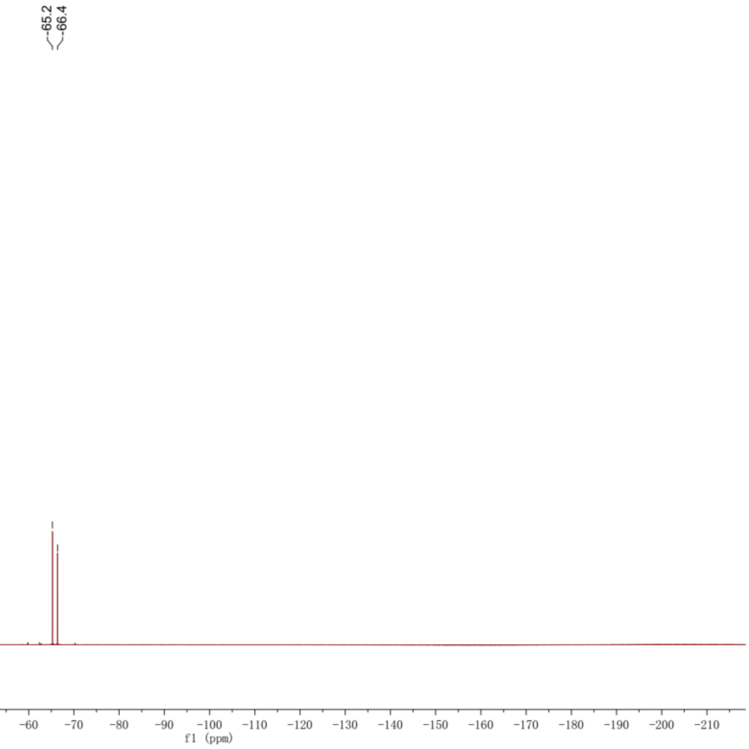

Supplement: Supplementary file 1 [file ol6c01481_si_001.pdf]
